# Co-expression and Transcriptome Analysis of *Marchantia polymorpha* Transcription Factors Supports Class C ARFs as Independent Actors of an Ancient Auxin Regulatory Module

**DOI:** 10.3389/fpls.2018.01345

**Published:** 2018-10-01

**Authors:** Eduardo Flores-Sandoval, Facundo Romani, John L. Bowman

**Affiliations:** ^1^School of Biological Sciences, Monash University, Melbourne, VIC, Australia; ^2^Facultad de Bioquímica y Ciencias Biológicas, Centro Científico Tecnológico CONICET Santa Fe, Instituto de Agrobiotecnología del Litoral, Universidad Nacional del Litoral – CONICET, Santa Fe, Argentina

**Keywords:** auxin, co-expression, *Marchantia*, auxin response factors, reproductive transitions, class C ARFs, transcriptome, miR160

## Abstract

We performed differential gene expression (DGE) and co-expression analyses with genes encoding components of hormonal signaling pathways and the ∼400 annotated transcription factors (TFs) of *M. polymorpha* across multiple developmental stages of the life cycle. We identify a putative auxin-related co-expression module that has significant overlap with transcripts induced in auxin-treated tissues. Consistent with phylogenetic and functional studies, the class C ARF, Mp*ARF3*, is not part of the auxin-related co-expression module and instead is associated with transcripts enriched in gamete-producing gametangiophores. We analyze the Mp*arf3* and Mp*miR160* mutant transcriptomes in the context of coexpression to suggest that Mp*ARF3* may antagonize the reproductive transition via activating the Mp*MIR11671* and Mp*MIR529c* precursors whose encoded microRNAs target *SQUAMOSA-PROMOTER BINDING PROTEIN-LIKE* (SPL) transcripts of Mp*SPL1* and Mp*SPL2*. Both Mp*SPL* genes are part of the Mp*ARF3* co-expression group corroborating their functional significance. We provide evidence of the independence of Mp*ARF3* from the auxin-signaling module and provide new testable hypotheses on the role of auxin-related genes in patterning meristems and differentiation events in liverworts.

## Introduction

Transcriptome studies in model systems provide a foundation to characterize genetic pathways in the absence of mutational studies. Transcriptome co-expression studies can highlight how specification of complex phenotypes depends on the activity of coordinated batteries of regulatory genes. In plants, many co-expression analyses focused on secondary metabolite production (Ruprecht et al., 2011; Ruprecht and Persson, 2012; Cassan-Wang et al., 2013; Ferreira et al., 2016; Turco et al., 2017), although co-expressed modules for hormonal genes (Ruprecht et al., 2017b; Singh et al., 2017), or developmental factors have also been described (Ichihashi et al., 2014). The functional significance of co-expression modules has been tested by further differential gene expression (DGE), protein-protein interaction (Kemmeren et al., 2002; Ichihashi et al., 2014), epigenetic (Turco et al., 2017) or functional analyses (Yokoyama et al., 2007; Ruprecht et al., 2011; Cassan-Wang et al., 2013; Ichihashi et al., 2014). Furthermore, studies have corroborated conservation of sets of co-expressed genes in closely related species (Ficklin and Feltus, 2011; Ichihashi et al., 2014; Netotea et al., 2014; Ferreira et al., 2016) as well as in deep evolutionary time (Stuart et al., 2003; Gerstein et al., 2014; Ruprecht et al., 2017a). From a simple ancestral genetic network, gene/genome duplication events could trigger the formation of novel co-expression clusters that can be re-wired and co-opted to pattern novel biological processes (Ruprecht et al., 2017b).

**FIGURE 1:**
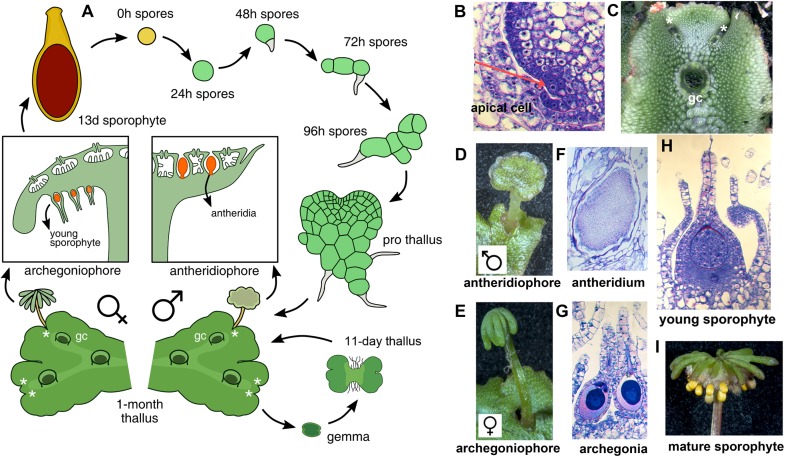
The life cycle of *M. polymorpha*. **(A)** Life cycle schematic of *M. polymorpha* displaying representative tissues of transcriptome libraries used in the study. Prothallus diagram based on Kny (1890). **(B)** Wild type thallus apex cross-section showing putative apical cell (red arrow). **(C)** Wild-type thallus showing dichotomous branching from apical notches. **(D)** Wild-type antheridiophore. **(E)** Wild-type archegoniophore. **(F)** Wild-type antheridia. **(G)** Wild-type archegonia. **(H)** Young wild-type sporophyte at similar to the sequenced 13-day sporophytes from Frank and Scanlon (2015). **(I)** Mature wild-type sporophytes protruding from archegoniophore. Asterisks = apical notches and gc = gemmae cups.

With the establishment of model bryophytes, an increasing number of transcriptome datasets describing multiple developmental and environmental conditions in mosses (Devos et al., 2016; Ortiz-Ramírez et al., 2016; Perroud et al., 2018) and liverworts (Alaba et al., 2015; Frank and Scanlon, 2015; Higo et al., 2016), are providing novel avenues to perform reverse genetic studies in non-vascular plant lineages. *Marchantia polymorpha* is a dioecious liverwort model system (Ishizaki et al., 2016) amenable to gene editing (Sugano et al., 2014) and gene silencing (Flores-Sandoval et al., 2016) techniques. Regulatory genes encoded in the *M. polymorpha* genome exhibit little genetic redundancy, with many transcription factor (TF) families represented by single paralogs (Bowman et al., 2017), making it an attractive system to characterize gene families that display genetic redundancy in other systems. The life cycle of *M. polymorpha* is haploid dominant (**Figure 1A**), with the diploid generation dependent upon the maternal haploid plant. Haploid spores germinate producing a sporeling, a developmental stage with active cell proliferation that forms a simple polarity between anchoring rhizoids and photosynthetic cells (Shimamura, 2016). In accordance with previous work (Bowman et al., 2017; Boehm et al., 2017), day 1 sporelings are single celled but actively differentiate chloroplasts, day 2 sporelings initiate a rhizoid cell, in day 3 sporelings the rhizoid cell elongates and the first divisions of the shoot occur, clearly establishing anatomical apical-basal polarity. By day 4, sporelings undergo proliferation of photosynthetic shoot tissue and further elongation of the rhizoid (**Figure 1A**). Using light cues, sporelings transition into prothalli, a two-dimensional heart-shaped body in which a meristematic zone with an apical cell producing derivatives in two planes is established (Flores-Sandoval et al., 2015). When the apical cell (**Figure 1B**) transitions to producing derivatives in four planes (top, bottom and lateral planes) a three-dimensional vegetative thallus (**Figure 1C**) is formed. Growth in thalli occurs at apical regions with differentiated tissues produced from apical cell derivatives and the apical meristem dichotomously branching (**Figure 1C**) at regular intervals (Shimamura, 2016; Solly et al., 2017). Upon inductive far-red to red light ratios (Kubota et al., 2014; Inoue et al., 2016; Linde et al., 2017) apices differentiate umbrella-like sexual gametangiophores (**Figures 1D,E**) wherein gametes (**Figures 1F,G**) are produced (Higo et al., 2016; Koi et al., 2016; Rövekamp et al., 2016; Yamaoka et al., 2018). In an moist environment, a sperm cell swims and fertilizes an egg cell, thus forming a zygote that undergoes several rounds of cell proliferation (**Figure 1H**) before differentiating sporogenous (**Figure 1I**) tissue (Shimamura, 2016).

Auxin is a key phytohormone that triggers multiple aspects of land plant physiology and development in a context dependent fashion. Comparative analysis between charophycean algae and land plant genomes and transcriptomes indicates that the canonical F-box mediated auxin signaling pathway evolved in the land plant ancestor (Bowman et al., 2017). Surprisingly, charophycean algae are able to transcriptionally respond to exogenous auxin (Hori et al., 2014; Ohtaka et al., 2017; Romani, 2017; Mutte et al., 2018) even in the absence of specific TIR1 F-box receptors, AUX/IAAs with auxin-sensitive degron (II) domains or distinct class A and B ARFs (Bowman et al., 2017; Flores-Sandoval et al., 2018; Mutte et al., 2018). *M. polymorpha* possesses single paralogs of all the canonical auxin-signaling pathway genes, retaining the predicted ancestral embryophyte state (Flores-Sandoval et al., 2015; Kato et al., 2015; Bowman et al., 2017; Mutte et al., 2018). In *M. polymorpha*, the class A ARF (Mp*ARF1*) is necessary to elicit physiological and transcriptional responses to exogenous auxin, acting as a transcriptional activator (Kato et al., 2017). The class B ARF (Mp*ARF2*) has not yet been characterized but is regarded as a transcriptional repressor (Kato et al., 2015). The class C ARF (Mp*ARF3*) is a target of the embryophyte-specific *miRNA160* and antagonizes differentiation events in multiple developmental contexts with loss-of-function Mp*arf3* alleles able to respond to exogenous auxin (Flores-Sandoval et al., 2018; Mutte et al., 2018), and strong gain-of-function Mp*ARF3* alleles resembling auxin-depleted mutants (Eklund et al., 2015; Flores-Sandoval et al., 2018). Phylogenetic analysis indicates Class C ARFs evolved in a charophycean algal ancestor prior to the emergence of the auxin-signaling module (Flores-Sandoval et al., 2018; Mutte et al., 2018). Two additional genes, the NON-CANONICAL ARF (Mp*NCARF*) and NON-CANONICAL IAA (Mp*NCIAA*), are predicted to form part of the auxin-mediated transcriptional response (Flores-Sandoval et al., 2018; Mutte et al., 2018). NCARFs evolved from a class A-like ARF in the embryophyte ancestor, and in *M. polymorpha* act synergistically with Mp*ARF1* to promote auxin signaling despite lacking a B3-DNA binding domain (Flores-Sandoval et al., 2018; Mutte et al., 2018). NCIAA genes form a sister clade to the AUX/IAA auxin signaling repressor genes, and as with the case of the class C ARFs, evolved prior to the origin of land plants (Flores-Sandoval et al., 2018; Mutte et al., 2018). Mp*nciaa* mutants are also sensitive to exogenous auxin (Mutte et al., 2018) suggesting they are not essential for auxin signaling. Despite conservation of these components across land plants, it is not known whether these genes act in common co-expression groups and whether their co-expression reflects functional dependency. Furthermore, the low number of annotated TFs, aMIR precursors and hormone-related genes in *M. polymorpha* allow accessible computation of a regulatory co-expression matrix, which perhaps is difficult in angiosperm model systems due to their high genetic redundancy. In this study we create a preliminary expression landscape of TFs defining developmental transitions in *M*. *polymorpha* based on available transcriptomic data. With co-expression analysis, we define gene clusters related to different tissues and developmental stages. Moreover, we reveal a cluster of auxin-related genes that are validated by significant overlap with auxin-inducible transcripts. We further show that the class C auxin response factor is expressed independently of this cluster and may negatively influence reproductive transitions in *M*. *polymorpha*.

## Materials and Methods

### DGE Expression Analysis

Fastq files of publicly available Illumina RNA-Seq libraries were obtained from the NCBI (SRA) depository. All libraries used for DGE analysis were performed in triplicates (except for antheridia – duplicates) and pairwise-comparisons were exclusively made with their original controls. *Marchantia* genome assembly (V3.1) and annotated gene models were used as references for TopHat2 mapping (Kim et al., 2013; Afgan et al., 2015). Control, Mp*arf3* and Mp*mir160* transcriptomes were sequenced and processed as previously described (Flores-Sandoval et al., 2018). DGE analysis was performed with edgeR and outputs presented were limited to curated TFs (**Supplementary Table S1**; Bowman et al., 2017) with logCPM > 0 and *P*-Values < 0.01. Box plots were generated using and online R application available at http://shiny.chemgrid.org/boxplotr/. Accession numbers from the NCBI Sequence Read Archive include: 11-day thalli (DRR050343, DRR050344, and DRR050345), Archegoniophore (DRR050351, DRR050352, and DRR050353), Antheridiophore, (DRR050346, DRR050347, and DRR050348), Antheridia (DRR050349, and DRR050350), apical cell (SRR1553294, SRR1553295, and SRR1553296), 13d-Sporophyte (SRR1553297, SRR1553298, and SRR1553299), Sporelings 0 h (SRR4450262, SRR4450261, and SRR4450260), 24hr-Sporeling (SRR4450266, SRR4450265, and SRR4450259), 48hr-Sporeling (SRR4450268, SRR4450264, and SRR4450263), 72hr-Sporeling (SRR4450267, SRR4450258, and SRR4450257), 96hr-Sporeling (SRR4450256, SRR4450255, and SRR4450254), 1-month wild type (SRR6685782, SRR6685783, and SRR6685784), 1-month Mp*arf3* (SRR6685778, SRR6685779, and SRR6685785), 1-month Mp*mir160* (SRR6685777, SRR6685780, and SRR6685781), thalli-mock (SRR5905100, SRR5905099, and SRR5905098), and 2,4-D 1-h (SRR5905097, SRR5905092, and SRR5905091). Alternatively, we performed DGE analysis with DESeq2 (Love et al., 2014) when stated in the text.

### Co-expression Analysis

Pearson’s correlations were calculated for all *Marchantia* TFs and for genes with putative connection with auxin biology: correlation coefficients were calculated amongst TFs and whole genome co-expression partners were calculated only for auxin-related genes. Gene sets were generated considering those with correlation > 0.8 and *P*-value < 0.001 as significantly co-expressed. Venn and Euler plots were generated using R package VennDiagram as well as VENNY (Oliveros, 2007). UpSet plots were generated using its respective R package (Lex et al., 2014). Heatmaps for TF co-expression matrixes were generated using Heatmapper (Babicki et al., 2016) by applying average linkage clustering to rows and columns and Euclidian distance measurements.

### Enrichment Analysis

Comparison of co-expression groups with differentially expressed genes were performed by enrichment analysis using Fisher’s exact test. For the auxin enrichment analysis we manually selected auxin related co-expression groups (and unrelated genes as negative controls) as a bait against: Auxin inducible genes from *Physcomitrella patens* (p.adjust < 0.05; Lavy et al., 2016), *Arabidopsis thaliana* (p.adjust < 0.01; Omelyanchuk et al., 2017) and *M. polymorpha* (p.adjust < 0.05; Mutte et al., 2018). For the first two species listed we determined orthologs using Phytozome^1^. -log_10_ *P*-values were reported in each Figure.

### GO and Protein Family Enrichment

Gene sets were functionally characterized via GO term enrichment analysis using an in-house Fisher’s exact test algorithm implemented in R (v3.3.2). We compiled Biological Process related GO term annotation for *M. polymorpha* genes using annotations available in Phytozome for *M. polymorpha* and *A. thaliana* and *P. patens* orthologs. We included in our GO term database both one-to-many and many-to-one relationships in order to obtain a more accurate annotation. For protein families, we used the same algorithm based on a different database. In this case, we annotated protein families using the HMMscan algorithm implemented in HMMER3 (Wheeler and Eddy, 2013) over the *M. polymorpha* proteome with default parameters and using Pfam profiles^2^.

## Results

### Transcription Factors Controlling Developmental Transitions in *M. polymorpha*

**FIGURE 2:**
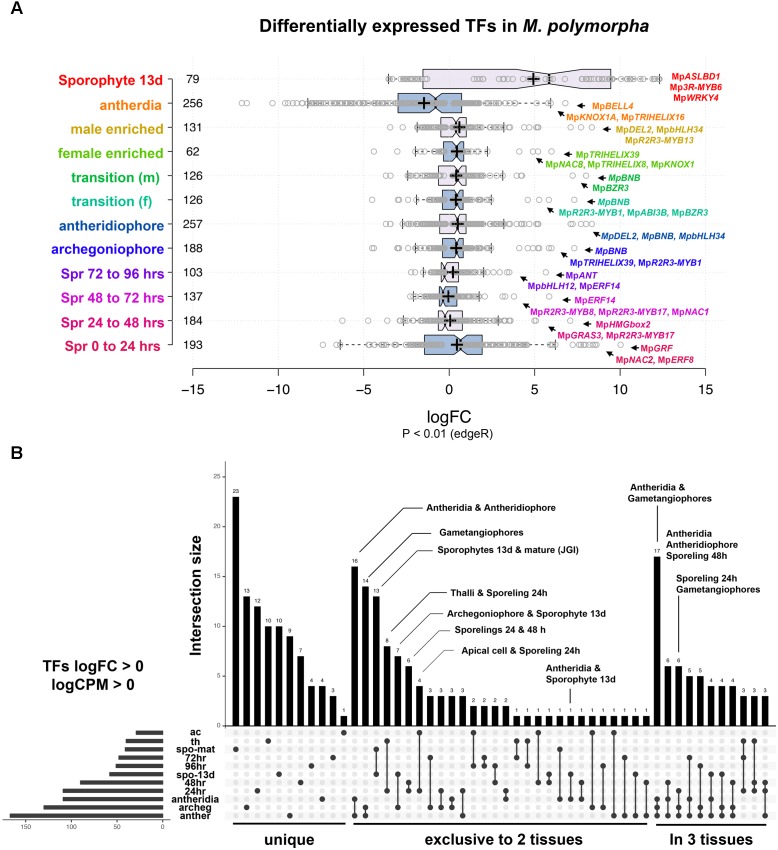
Differential gene expression analysis in *M. polymorpha.* **(A)** Boxplots of log_2_FC value distribution of differentially expressed (edgeR, *P* < 0.01) TFs in multiple pair-wise comparisons. Central lines represent medians; box limits indicate the 25th and 75th percentiles as determined by R software (http://shiny.chemgrid.org/boxplotr/); whiskers extend 1.5 times the interquartile range from the 25th and 75th percentiles, outliers are represented by dots; crosses represent sample means; data points are plotted as open circles. Number of sample points are included for each group. **(B)** UpSet diagram indicating intersections between upregulated TFs (*P* < 0.01, logFC > 0) in multiple pairwise comparisons. Diagram indicates uniquely up-regulated TFs on the left, followed by those exclusively shared between two and three pairwise comparisons. Abbreviations: ac, apical cell; th, thallus; spo-mat, JGI-sporophytes; 72 h, 72-h sporelings; 96 h, 96-h sporelings; spo-13 d, 13-day sporophytes; 48 h, 48-h sporelings; 24 h, 24-h sporelings; archeg, archegoniophores; anther, antheridiophores.

We performed DGE analysis on publicly available RNA-Seq libraries to (1) identify transcription factors defining or influencing *Marchantia* developmental transitions and (2) use the obtained datasets as references for enrichment analysis (Frank and Scanlon, 2015; Higo et al., 2016; Bowman et al., 2017). Datasets span multiple tissues throughout the life cycle, including intensively sampled sporelings during the first 5 days of development (Bowman et al., 2017), apical cells (Frank and Scanlon, 2015), gametangiophores, antheridia (Higo et al., 2016), and 13-day old sporophytic micro-dissections (Frank and Scanlon, 2015). In our analysis, 82% of differentially expressed TFs with *P* < 0.01 (edgeR) have fold changes (FC) below 4× in most tissues except sporophytes (**Figure 2A** and **Supplementary Figure S1A**), suggesting feedback regulation preventing drastic changes in regulatory gene expression. To account for both read abundance and fold-changes, we used the product of logFC and logCPM (counts per million) to categorize the transcripts enriched in a particular tissue. Of the 405 TFs in the *M. polymorpha* genome (**Supplementary Table S1**), 45 were not differentially expressed in any of the performed pair-wise comparisons (**Supplementary Figure S1B**).

In order to find TFs specific to a developmental transition, we compared all upregulated TFs in each tissue using a cut off of logFC and logCPM > 0 (**Figure 2B** and **Supplementary Table S2**). Mature sporophyte-enriched TFs sequenced by JGI (Bowman et al., 2017) were included in this comparison to validate the 13-day microdissected sporophytes with both datasets showing a high degree of overlap (**Figure 2B**). Mature sporophytes have the largest number of uniquely upregulated TFs (**Figure 2B**), followed by 24-h germinated spores, gametangiophores and thalli. The apical cell and antheridia had a single uniquely upregulated TF each (**Figure 2B**). As expected, antheridia and antheridiophores, male and female gametangiophores and thalli/24-h sporelings show the highest degree of similarity. Surprisingly, 13-day sporophytes, but not mature sporophytes, had common and exclusive upregulation with archegoniophores and antheridia. Finally, antheridia, antheridiophores and archegoniophores formed a large group of uniquely upregulated TFs (**Figure 2B**). The TFs enriched in each tissue library will be described in detail in the following paragraphs.

#### Sporelings 0 to 24 h

**FIGURE 3:**
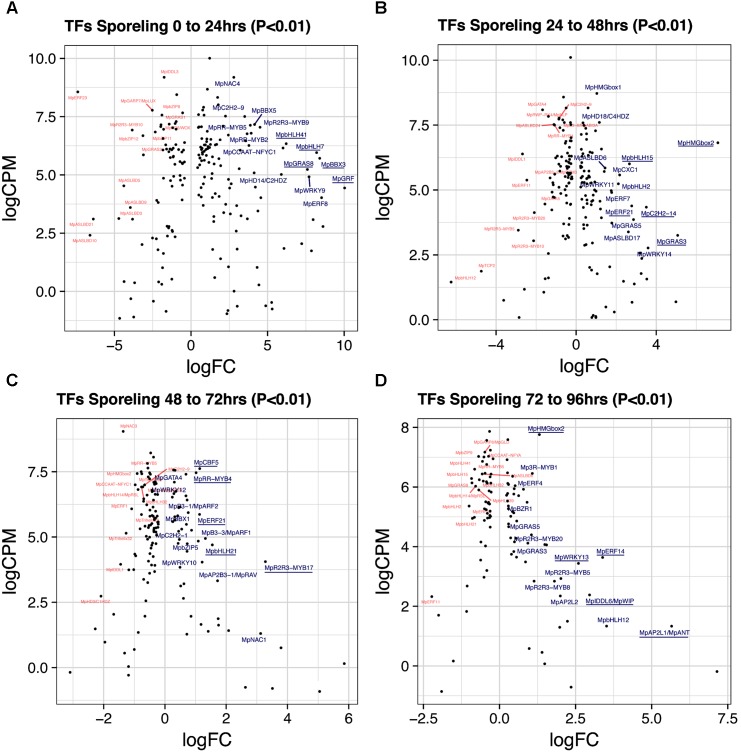
Differentially expressed TFs in *M. polymorpha* sporelings. LogFC and logCPM outputs of DGE analysis showing pairwise comparisons of **(A)** 0 vs. 24 h, **(B)** 24 vs. 48 h, **(C)** 48 vs. 72 h, and **(D)** 72 vs. 96 h. Top 15 upregulated (blue) and downregulated (red) TFs are annotated, with the top5 upregulated TFs underlined.

A total of 193 TFs (114 upregulated) define the transition of dormant spores to germinated ones (**Figure 2A** and **Supplementary Table S3**). This stage has the third highest number of upregulated TFs after gametangiophores (logFC > 0), and has the highest number of upregulated TFs at logFC > 2 (**Supplementary Figure S1A**). This likely reflects the large number of physiological, cellular and developmental processes being activated upon germination. The ortholog of the root specifying *TARGET OF MONOPTEROS 5/ABNORMAL SHOOT 5* (Schlereth et al., 2010), Mp*bHLH7* (Mapoly0039s0068, logFC = 8 and logCPM = 5.9) is the highest expressed TF incorporating both fold-changes and read abundance in 1-day sporelings (**Figure 3A**). Mp*bHLH7* is followed by the B-box type ZINC FINGER gene Mp*BBX3* (Mapoly0049s0067, logFC = 8.4 and logCPM = 5.7) and the single *M. polymorpha* GROWTH REGULATING FACTOR gene Mp*GRF* (Mapoly1350s0001, logFC = 10 and logCPM = 4.4). Mp*GRAS8* (Mapoly0065s0089, logFC = 7.6 and logCPM = 5.23) from an uncharacterized GRAS lineage in plants (Bowman et al., 2017), and Mp*bHLH41* (Mapoly0100s0033, logFC = 6.21 and logCPM = 6.23), an ortholog of NAI1, a TF involved in activation of post-germinative seedling programs in *Arabidopsis* (Yoshii et al., 2015) are also highly upregulated. An auxin-inducible gene (Mutte et al., 2018), the HOMEODOMAIN class II HD-ZIP, Mp*C2HD*Z (Mapoly0069s0069, logFC = 5.89 and logCPM = 5.01) also has high expression consistent with up-regulation of Mp*YUC2* (Mapoly0063s0040, logFC = 1.32 and logCPM = 3.55). Co-expression dynamics for day-1 sporeling enriched genes shows Mp*bHLH7* and Mp*C2HD*Z are highly correlated. Meanwhile Mp*GRF* expression correlates with that of the class I TCP Mp*TCP1*, the auxin signaling repressor Mp*IAA* and the HOMEODOMAIN class IV HD-ZIP Mp*C4HDZ* (**Supplementary Figure S2**).

#### Sporelings 24 to 48 h

A total of 184 TFs (90 upregulated, **Supplementary Table S4**) define the second day of sporeling development where the first rhizoid is formed (Bowman et al., 2017). Mp*HMGbox2* (Mapoly0026s0138, logFC = 7 and logCPM = 6.8) and Mp*HMGbox1* (Mapoly0013s0048, logFC = 1.03 and logCPM = 8.71) are upregulated and abundant (**Figure 3B**). They are orthologs of the 3xHIGH MOBILITY GROUP-BOX2 genes involved in cell cycle regulation (Pedersen et al., 2011). These are followed by two GRAS TFs: Mp*GRAS3* (Mapoly0014s0183, logFC = 5 and logCPM = 3.2) and Mp*GRAS5* (Mapoly0047s0034, logFC = 2.86 and logCPM = 3.85), which belong to separate and uncharacterized GRAS lineages in land plants (Bowman et al., 2017). Mp*ERF21* (Mapoly0166s0010, logFC = 2.77 and logCPM = 4.38), an ABA REPRESSOR1 (ABR1) ortholog and Mp*ERF7* (Mapoly0034s0029, logFC = 1.77 and logCPM = 4.9) a DREB26 ortholog, are two AP2/ERF TFs enriched in this transition and putatively involved in stress-responses (Pandey, 2005; Krishnaswamy et al., 2011). Finally, Mp*bHLH15* (Mapoly0038s0058, logFC = 2 and logCPM = 6), an ortholog of CRYPTOCHROME-INTERACTING (CIB) bHLHs (Liu et al., 2008) is also enriched in day two sporelings (**Figure 3B**). Co-expression dynamics for day-2 sporeling enriched genes shows Mp*HMGbox1* and *2* form a distinct group, while Mp*ERF7* and *21* are co-expressed (**Supplementary Figure S3**).

#### Sporelings 48 to 72 h

A total of 142 TFs (50 upregulated, **Supplementary Table S5**) define this transition, where photosynthetic vs. anchoring tissue polarity is properly established (Bowman et al., 2017). Mp*R2R3-MYB17* (logFC = 3.2, logCPM = 4.05) a MIXTA-like MYB TF is the highest expressed gene after incorporating abundance (**Figure 3C**). It belongs to a MYB lineage (clade IV) composed of genes regulating secondary compound metabolism (Bowman et al., 2017). Similar to day 2, Mp*ERF21* continues its enrichment (logFC = 1.12 and logCPM = 5.86), as well as Mp*bHLH21* (Mapoly0037s0007, logFC = 1.54 and logCPM = 4.69) a paralog of Mp*bHLH15* (CIB bHLHs). The auxin signaling pathway, represented by Mp*ARF1* (Mapoly0019s0045, logFC = 1.32 and logFC = 4.94) and Mp*ARF2* (Mapoly0011s0167, logFC = 0.72 and logFC = 6.41) are significantly upregulated in this transition (**Supplementary Figure S4A**). The CUP-SHAPE COTYLEDON ortholog Mp*NAC1* (Mapoly0015s0058, logFC = 3.1 and logCPM = 1.3) and the RAV-like B3-domain TF Mp*RAV* (Mapoly0072s0102, logFC = 1.71 and logCPM = 3.32) are also enriched. The logFC average in 3-day sporelings has decreased by ∼1/2 (**Figure 2A**) from 1-day sporelings, suggesting increased homeostatic control of TF expression. Mp*R2R3-MYB17*, Mp*ARF1*, Mp*ARF2*, and Mp*NAC1* form a robust co-expression cluster (**Supplementary Figure S5**).

#### Sporelings 72 to 96 h

A total of 106 TFs (52 unregulated, **Supplementary Table S6**) define this transition, where there is a proliferation of photosynthetic cells and elongation of the rhizoid (Bowman et al., 2017). The *ANT/PLETHORA*/*BABYBOOM* ortholog Mp*ANT* (Mapoly0008s0071, euANT clade) involved in meristematic activity in mosses (Aoyama et al., 2012) and angiosperms (Aida et al., 2004; Kareem et al., 2015) is the TF with the highest fold-change (logFC = 5.6) despite being mildly expressed (logCPM = 1.3, **Figure 3D**). The auxin-induced gene Mp*WIP* (Mapoly0096s0050, logFC = 2.95 and logCPM = 2.38) necessary for air pore development (Jones and Dolan, 2017) follows a similar pattern. Mp*ERF14* (Mapoly0066s0103, logFC = 3.38 and logCPM = 3.63) from clade VIII of the ERF phylogeny (Bowman et al., 2017), Mp*HMGbox2* (logFC = 7.75 and logCPM=) and Mp*WRKY13* (Mapoly0162s0003, logFC = 2.59 and logCPM = 3.43) are highly expressed and upregulated (**Figure 3D**). The auxin-signaling TFs Mp*ARF1* (logFC = 0.46 and logCPM = 5.76) and Mp*ARF2* (logFC = 0.24 and logCPM = 6.91) continue to be highly expressed and upregulated (**Supplementary Figure S4A**). Enrichment analysis (Fishers-exact test) probing auxin-induced *M. polymorpha* transcripts (Mutte et al., 2018) with sporeling and thalloid transcriptomes shows that 96-h sporelings vastly converge with genes activated by auxin, perhaps as a result of progressive increases of class A and B ARF activity (**Supplementary Figure S4B**). All of the above mentioned genes form a robust co-expression cluster (**Supplementary Figure S5**).

#### Thalli vs. Archegoniophores

**FIGURE 4:**
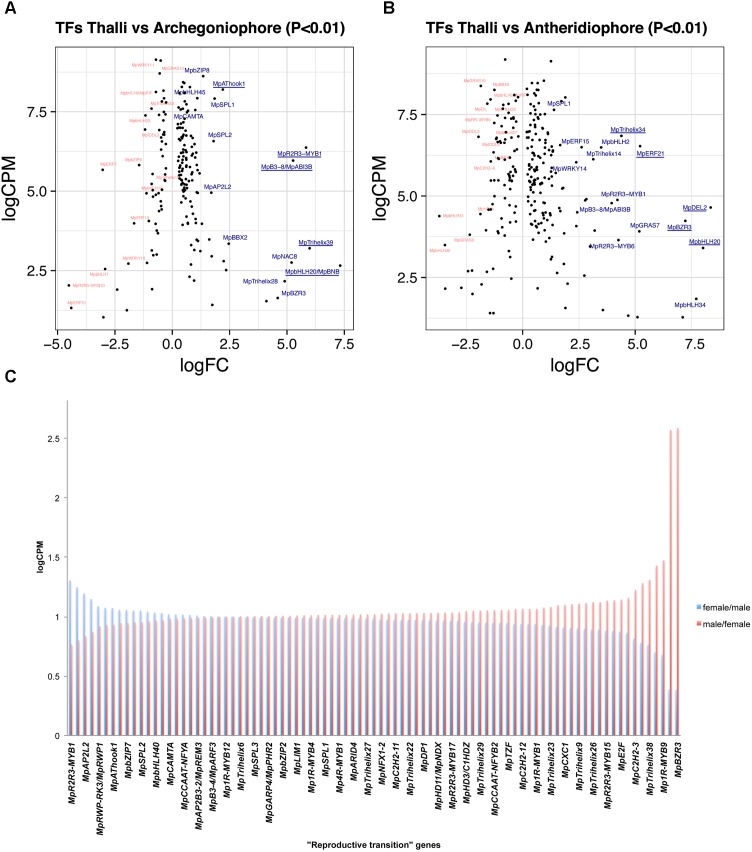
Differentially expressed TFs in *M. polymorpha* gametangiophores. LogFC and logCPM outputs of DGE analysis showing pairwise comparisons of **(A)** thalli vs. archegoniophores and **(B)** thalli vs. antheridiophores. Top 15 upregulated (blue) and downregulated (red) TFs are annotated, with the top5 upregulated TFs underlined. **(C)** TFs upregulated in both sexes were defined as reproductive transition genes, most of which have 1/1 ratios of logCPM values in males and females.

A total of 188 TFs (129 upregulated, **Supplementary Table S7**) are differentially expressed between 11-day thalli and mature archegoniophores (Higo et al., 2016). The TFs with highest expression at this stage (**Figure 4A**) is the bHLH factor Mp*BONOBO/MpBNB* (Mapoly0024s0106, logFC = 7 and logCPM 2.6), which has been shown to promote formation of gametangiophores and biogenesis of gametes (Yamaoka et al., 2018). Mp*R2R3-MYB1* (Mapoly0001s0061) is a highly expressed and upregulated factor in archegoniophores (logFC = 5.26 and logCPM = 6.37), followed by the B3-domain Mp*ABI3B* (Mapoly0474s0001, logFC = 5.26 and logCPM = 5.96) whose orthologs in *Arabidopsis* modulate abscisic acid responses (Mccarty et al., 1991; Suzuki et al., 2001). Finally, the putative GT2-like Mp*TRIHELIX39* (Mapoly0003s0229, logFC = 5.98 and logCPM 3.2) has drastic fold changes but lower read counts (**Figure 4A**).

#### Thalli vs. Antheridiophores

A total of 257 TFs (166 upregulated, **Supplementary Table S8**) are differentially expressed in antheridiophores when compared to 11-day thalli. One of the two DP-E2F-LIKE PROTEIN TFs in *Marchantia*, Mp*DEL2* (Mapoly0198s0008, logFC = 8.34 and logCPM = 4.64), involved in regulating the cell cycle and cell proliferation (Sozzani et al., 2010), as well as Mp*BNB* (logFC = 8 and logCPM = 3.4) are the TFs with the highest fold change in the transition from thalli to antheridiophores (**Figure 4B**). In addition, Mp*ERF21* (Mapoly0166s0010, logFC = 5.14 and logCPM = 6.52), Mp*BZR3* (Mapoly0072s0031, logFC = 7.22, logCPM = 4.23), and Mp*TRIHELIX34* (Mapoly0159s0017, logFC = 4.38, logCPM = 6.84) are highly expressed genes with large fold-changes in antheridiophores (**Figure 4B**).

#### Reproductive Transition

A total of 126 TFs (87 upregulated, **Supplementary Table S9**) are shared with similar dynamics in thalli vs. archegoniophore and thalli vs. antheridiophore comparisons (**Figure 4C**). Nine of these genes were enriched in no other tissue but gametangiophores albeit being mildly upregulated (**Figure 2B** and **Supplementary Table S2**). Thirteen genes were exclusive to gametangiophores and antheridia, corroborating their specificity in reproductive roles (**Figure 2B** and **Supplementary Table S2**). Mp*BNB* is in this former group, consistent with its role in both gametangiophore and gamete formation (Yamaoka et al., 2018). Additional genes were enriched but not specific to gametangiophores (**Supplementary Table S2**). A majority of these TFs (**Figure 4C**) have 1:1 ratios of logCPM between male and female tissues with only Mp*R2R3-MYB1* being higher in females (logCPM female/male = 1.3) and Mp*BZR3* and Mp*R2R3-MYB6* preferentially expressed in males (logCPM male/female = 2.5 in both cases). Despite their high expression in males, complete Mp*R2R3-MYB1* transcripts were exclusively found in females, suggesting male expression may be represented by incomplete transcripts.

#### Female Enriched Genes

**FIGURE 5:**
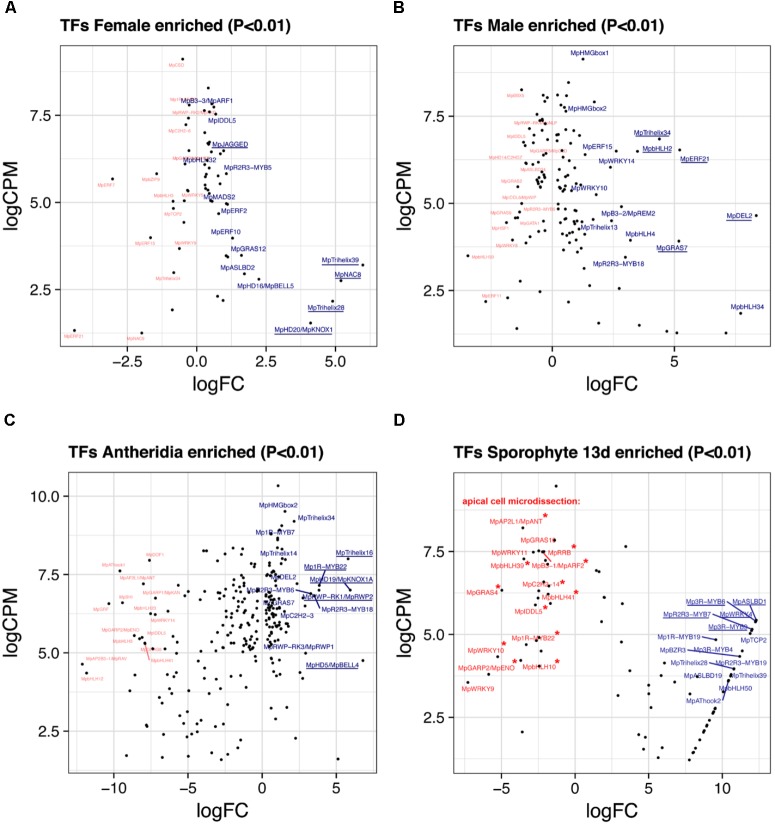
Sex-enriched and sporophyte TFs obtained from DGE analysis. LogFC and logCPM outputs of DGE analysis showing TFs solely enriched in **(A)** archegoniophores and not antheridiophores, **(B)** antheridiophores and not archegoniophores, **(C)** antheridia vs. antheridiophores, and **(D)** 13-day sporophytes vs. apical cell RNAs. Top 15 upregulated (blue) and downregulated (red) TFs are annotated, with the top5 upregulated TFs underlined. Asterisks in **(D)** represent TFs not upregulated in thallus tissue.

Due to the lack of archegonia-specific transcriptomes, we defined female enriched genes by discarding reproductive transition genes from the thalli vs. archegoniophore comparison (62 genes in total, 42 upregulated, **Supplementary Table S10**). These include (**Figure 5A**) Mp*NAC8* (Mapoly0035s0054, logFC = 5.19 and logCPM = 2.75), which is an ortholog of LONG VEGETATIVE PHASE (LOV1) involved in repressing flowering transitions in *Arabidopsis* (Yoo et al., 2007); Mp*TRIHELIX39*, and the SH4-like Mp*TRIHELIX28* (Mapoly0122s0030, logFC = 4.89 and logCPM = 2.16). Additionally, two TALE-HOMEODOMAIN TFs are also upregulated in females although with lower read counts, possibly showing a diluted signal from archegonia RNA. These include the class I KNOX gene Mp*KNOX1* (Mapoly0175s0020, logFC = 4.1 and logCPM = 1.53) as well as a BELL gene, Mp*BELL5* (Mapoly0093s0028, logFC = 2.23 and logCPM = 2.79), the latter only having orthologs in charophycean algae (Bowman et al., 2017).

#### Male Enriched Genes

A total of 131 TFs (79 upregulated, **Supplementary Table S11**) are enriched in antheridiophores after discarding reproductive transition genes. Mp*DEL2* and Mp*TRIHELIX34* are kept in this category (**Figure 5B**). Additional genes include, Mp*ERF21* (Mapoly0166s0010, logFC = 5.21 and logCPM = 6.52), Mp*bHLH2* (Mapoly0028s0062, logFC = 3.48 and logCPM = 6.49) an ortholog of AT4G09820, Mp*ERF15* (Mapoly0068s0088, logFC = 2.61 and logCPM = 6.49), Mp*GRAS7* (Mapoly0064s0023, logFC = 5.18 and logCPM = 3.91) a gene lost in the common ancestor of seed plants (Bowman et al., 2017), and Mp*WRKY14* (Mapoly0467s0001, logFC = 2.37 and logCPM = 6.49) that belongs to clade III in the WRKY phylogeny (Bowman et al., 2017).

#### Antheridiophore vs. Antheridia

A total of 256 TFs (108 upregulated, **Supplementary Table S12**) are differentially expressed in antheridia when compared to antheridiophores. The higher ratio of repressed/activated TFs suggests the substitution of haploid photosynthetic and free-living thalli for that of more specialized gamete forming pathways (**Figure 2A**). For example, TFs upregulated during sporeling development, such as Mp*GRF* (logFC = -10) and Mp*ANT* (logFC = -8) are repressed (**Figure 5C**). Upregulated genes in antheridia in both in terms of fold-change and abundance (**Figure 5C**) are an ortholog of the SH4-like Mp*TRIHELIX16* (Mapoly0037s0146, logFC = 5.79 and logCPM = 7.99); Mp*KNOX1A* (Mapoly0174s0007, logFC = 5.93 and logCPM = 7); and Mp*KNOX1B* (Mapoly0023s0081, logFC = 2.55 and logCPM = 4.37), which are semi conserved TALE (KNOX1) HD genes that have lost the DNA binding domain (Bowman et al., 2017), Mp*BELL4* (Mapoly0013s0027, logFC = 6.79 and logCPM = 4.75) and Mp*BELL5* (logFC = 2.25 and logCPM = 2.27). Mp*BELL4* in particular is not detected as differentially expressed in thalli vs. antheridiophores, suggesting it is male-gamete specific, while Mp*BELL5* shows incomplete transcription in males, suggesting that it might be a female-associated factor. Additional genes previously curated (Higo et al., 2016) include Mp*1R-MYB22* (Mapoly0811s0001, logFC = 3.85 and logCPM = 7.15), Mp*RWP2/MpMID* (Mapoly0014s0044, logFC = 3.83, logCPM = 7) whose ortholog in the chlorophycean algae *Chlamydomonas* specifies *minus* gametes (Ferris and Goodenough, 1997; Lin and Goodenough, 2007), and Mp*R2R3-MYB18* (Mapoly0123s0012, logFC = 3.52 and logCPM = 6.81) an ortholog of FOUR LIPS (FLP), which restricts mitosis in stomatal development in *Arabidopsis* (Lee et al., 2013).

#### Sporophytic Genes

We re-analyzed the microdissected RNA of 13-day-old sporophytes given that at the time of publication (Frank and Scanlon, 2015) there was no annotated reference genome. These samples have low read-mapping rate using TopHat2 (∼60%), which would leave differentially expressed genes with low CPM undetected. Replicates were compared to its endogenous control, the apical notch microdissected libraries. Due to these limitations, the 85 differentially expressed TFs (57 upregulated, **Supplementary Table S13**) in sporophytes are skewed to high-fold changes (**Figures 2A**, **5D**). The TFs shared between 13-day sporophytes and mature JGI sporophyte libraries (**Figure 2B**) include the AP2/B3 domain Mp*REM4* (Mapoly0006s0205, logFC = 13.86 and logCPM = 6.96), several FAMA-like orthologs Mp*bHLH35-37* and *50* (Mapoly0031s0072, 74, 75, and 76; average logFC = 9.4 and logCPM = 2.67) involved in stomata development in mosses and tracheophytes (Ohashi-Ito and Bergmann, 2006; Chater et al., 2016); multiple Mp*3R-MYBs* (Mp*3R-MYB3-6* and *9*) among others (**Supplementary Table S13**). However, representative mature sporophyte-enriched genes such as the TALE-class HOMEODOMAIN Mp*BELL1* (Mapoly0213s0014), Mp*KNOX2* (Mapoly0194s0001) and the AP2/B3 domain Mp*REM1* (Mapoly0183s0010) were not upregulated in 13-day sporophytes (**Supplementary Table S13**). Enrichment analysis confirmed significant overlap between differentially expressed JGI and 13-day sporophyte transcriptomes with a slight significant overlap between female-enriched genes and 13-day sporophytes (**Supplementary Figure S6A**). Female enriched TFs (**Figure 5A**) upregulated in 13-day sporophytes include Mp*TRIHELIX28* and *39*, Mp*ASLBD2*, Mp*KNOX1*, Mp*GRAS12*, Mp*NAC8*, and Mp*bHLH29* (**Figure 2B** and **Supplementary Table S2**). This could be due to archegonia RNA contamination or a continuation of maternal developmental programs in young sporophytes. Supporting the latter interpretation, 13 of previously classified antheridia enriched genes (**Figure 5C**) including Mp*BELL4*, Mp*TRIHELIX16*, Mp*R2R3-MYB3*, *6* and *13*, Mp*GRAS7* and Mp*DEL2* were also detected as upregulated in 13-day sporophytes (**Supplementary Figure S6B**). Mp*BELL4* is the only TF exclusively upregulated in antheridia and 13-day sporophytes in our analysis (**Supplementary Table S2**). Finally, Mp*BELL5* is upregulated in archegoniophores, antheridia (incomplete transcript) and 13-day sporophytes (**Supplementary Table S2**).

There are additionally seven genes uniquely upregulated in 13-day sporophytes (**Figure 2B**), these include: Mp*WRKY4* (Mapoly0031s0170), Mp*ASLBD19* (Mapoly0817s0001), Mp*AP2L5* (Mapoly0144s0032), among others (**Supplementary Table S2**). Orthologs of sporophyte-enriched *Physcomitrella* genes (Ortiz-Ramírez et al., 2016) include (**Supplementary Table S2**) the class II TCP TF Mp*TCP2* (Mapoly0001s0298, logFC = 11.89 and logCPM = 5.02), Mp*REM4*, Mp*ABI3B* (Mapoly0474s0001, logFC = 8.29 and logCPM = 3.72) and the SQUAMOSA-PROMOTER BINDING PROTEIN-LIKE Mp*SPL1* (Mapoly0014s0224, logFC = 1.44 and logCPM = 6.92). These genes are upregulated in other tissues and only Mp*REM4* seems to be sporophyte-specific (**Supplementary Table S2**).

#### Apical Cell

We used the microdissected putative apical cell libraries as controls for the sporophyte DGE analysis (Frank and Scanlon, 2015). This potentially shows that down-regulated genes in sporophytes are upregulated in apical cells (**Figure 5D**). To try to refine this comparison we discarded genes that were robustly (logFC > 1) upregulated in other tissues (**Supplementary Figure S7** and **Supplementary Table S14**). This resulted in 8 TFs exclusively enriched at logFC > 1 in apical cells. These included a GARP TF Mp*GARP2/ENO* (Mapoly0157s0009, logFC = 5.87 and logCPM = 3.79) belonging to a lineage sister to KANADI genes (Bowman et al., 2017), the B3-domain Mp*ARF2* auxin signaling transcriptional repressor (Mapoly0011s0167, logFC = 2.2 and logCPM = 7.48), the single member of the basal SCGJ bZIP group Mp*bZIP15* (Mapoly0737s0001, logFC = 2.13 and logCPM = 6.58) and 5 other genes (**Supplementary Table S4**). Outside this group, the highest expressed TF in the apical cell RNA is Mp*GRAS4* (Mapoly0031s0041, logFC = 4.97 and logCPM = 6.32), an ortholog of LATERAL SUPRESSOR involved in axillary meristem establishment in *Arabidopsis* (Greb et al., 2003). Mp*GRAS4* however, is strongly upregulated in 24 h-sporelings (logFC = 3.26 and logCPM = 3.24), 48 h-sporelings (logFC = 1.18) and weakly in antheridiophores (logFC = 0.89). Mp*ANT* is robustly enriched in apical cell tissue (logFC = 3.55 and logCPM = 8.21) but also in 96 h sporelings (**Figure 2A**). Four genes were exclusively upregulated in apical cells and 24 h-sporelings at logFC > 1: Mp*bHLH39* (Mapoly0073s0059, logFC = 3.48 and logCPM = 7.27), Mp*bHLH41*, the type-B RESPONSE REGULATOR Mp*RR-B* (Mapoly0101s0006) and Mp*WRKY9* (Mapoly0051s0057). Finally, Mp*WRKY10* (Mapoly0057s0012, logFC = 5.25 and logCPM = 4.32) is upregulated exclusively in apical cells and antheridiophores (**Supplementary Table S2**).

### *Marchantia* Regulatory Genes Are Organized in Co-expression Groups

**FIGURE 6:**
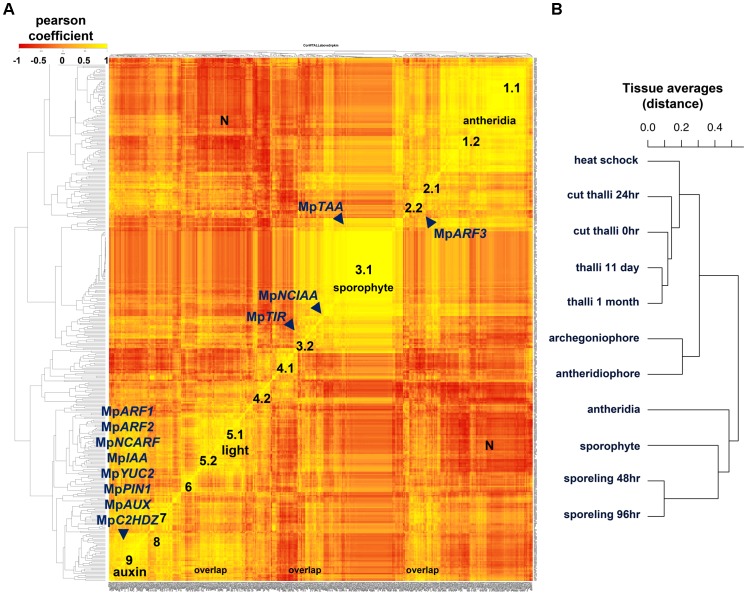
Co-expression matrix of regulatory genes in *M. polymorpha* **(A)** Heatmap of Pearson coefficient matrix of all annotated *M. polymorpha* TFs, MIR precursors and putative hormone response and synthesis pathway genes (**Supplementary Table S15**) in 11 tissue libraries using RPKM values > 5. Co-expression groups were delimited by sets of genes sharing coefficients above 0.8. Genes putatively involved in auxin biosynthesis, perception, signaling and transport are mapped. **(B)** Distance dendrogram of tissue libraries used in the analysis. Average replicate RPKM values were used per library. Scale for heatmap indicates Pearson coefficients.

In order to predict functional roles of clusters of TFs sharing similar expression dynamics, we created a co-expression matrix comparing averages of normalized reads per kilobase per million (RPKMs) across 11 different tissues. Pearson coefficients were calculated for each of the annotated *Marchantia* TFs, microRNA (miR) precursors as well as hormone biosynthetic and signaling genes. Two stages of sporeling development (48 and 96 h), multiple thallus RNAs, archegoniophores, antheridiophores, antheridia and older sporophytes (JGI) were used in this analysis. Using a minimal RPKM value of 5 as a threshold, a Pearson coefficient heatmap (**Figure 6A**) suggests at least 9 highly correlated groups, some of which show a greater degree of correlation than others. A distance dendrogram (RPKM < 1) supported the occurrence of multiple groups (**Supplementary Figure S8**) and we focused on describing nine of the most conspicuous in basis of their tissue-specificity or putative functional relationships (**Supplementary Figure S9**). The antheridia (group 1) and sporophyte-enriched genes (group 3) combined comprise ∼1/2 of the total sample. This is in agreement with these tissues being the most divergent in terms of form and physiology.

Group 1 defined by Mp*BELL4* partners (Pearson > 0.8, average coefficient = 0.91) composed of 47 TFs includes Mp*TRIHELIX16*, Mp*KNOX1A*, Mp*KNOX1B*, Mp*RWP2/MID*, Mp*1R-MYB22*, Mp*PIN2*, Mp*LFY*, Mp*GRAS7* among others (**Supplementary Figure S9A**). A diffuse group (group 2) includes 9 partners of Mp*ARF3* (Pearson > 0.8, average coefficient = 0.85), many of which were classified as reproductive transition genes, such as, Mp*SPL1-3*, Mp*bZIP*8, Mp*bHLH17* and *48*, Mp*RR-B*, Mp*C3HDZ*, among others (**Supplementary Figure S9B**). Sporophytic genes (group 3) can be divided between those enriched or specific to the sporophyte. The Mp*BELL1* subgroup (Pearson > 0.8 and average coefficient = 0.94) is composed of 96 genes including Mp*REM1* and *4*, Mp*KNOX2*, Mp*YUC1*, Mp*PYL2-4*, Mp*1R-MYB5,6,19 and 21*; Mp*3R-MYB2,4-9*; Mp*bHLH8, 9, 11, 18, 22, 25, 34-37* and *51;* Mp*ASLBD1,-5, 8, 9, 11, 13-16, 19 and 21*, most of which are exclusive to the mature sporophyte (**Supplementary Figure S9C**). Meanwhile, the Mp*NCIAA* subgroup (Pearson > 0.8, average coefficient = 0.84) is composed of 25 genes including Mp*TAA*, Mp*ERF17*, Mp*GRAS2*, Mp*LUX*, Mp*PHR2*, Mp*ACS2*, Mp*MIR319a*, Mp*IDDL1-2*, Mp*ASLBD2* and others that fit in the first category (**Supplementary Figure S9D**). A diffuse group 4.1 (Pearson > 0.8, average coefficient = 0.88) includes six partners of Mp*bHLH14*/Mp*RSL* (**Supplementary Figure S9E**), which has been shown to promote epidermal outgrowths (Proust et al., 2016). Members of this group include Mp*NAC4*, Mp*GRAS8*, and Mp*MIR11671* a negative regulator of Mp*SPL1* (Tsuzuki et al., 2015; Lin et al., 2016). Group 4.1 is expressed maximally in 48 h. sporelings and steadily decreases at later stages (**Supplementary Figure S9E**). Similar to group 4.1, Mp*GLD* forms a subgroup 4.2 (Pearson > 0.8, average coefficient = 0.91) with five other genes including Mp*C2H2-9*, Mp*bHLH32*, *41*, and Mp*RR-MYB5* that follows a similar decrease in expression pattern albeit with higher expression in the thallus (**Supplementary Figure S9F**). Group 5 is defined by Mp*TOC1* partners (Pearson > 0.8, average coefficient = 0.81), composed of 12 TFs including Mp*bHLH6*/Mp*PIF*, the Type-A RESPONSE REGULATOR Mp*RR-A*, Mp*BBX5*, Mp*WIP*, Mp*ETR1*, Mp*ERF10*, Mp*GRAS9* among others, possibly reflecting a light signaling/circadian clock module (Inoue et al., 2016; Linde et al., 2017). Group 5 has peaks in thalli with excised apical notches (0 h) and heat-shocked thalli (**Supplementary Figure S9G**). Mp*KAN* and its partners overlap with most of group 5 (Pearson > 0.8, average coefficient = 0.86) but form a larger group with 42 other members that include Mp*WRKY8* and *11*, Mp*bHLH15, 23, 27* and *31*, Mp*HSR*, and auxin-related genes (which will be discussed in the following paragraph) at the ∼20th rank. The Mp*KANADI* group has its average highest expression only at 0-h cut thalli with excised apical notches suggesting a role in differentiated tissues (**Supplementary Figure S9H**). Group 6 (**Supplementary Figure S9I**) comprises 10 genes highly expressed in regenerating thalli (after 24 h of apical notch excision) and includes partners of the apical-cell enriched Mp*GRAS4* (Pearson > 0.8. average coefficient = 0.85). Other partners include Mp*R2R3-MYB5* and *20*, Mp*ERF20*, Mp*bHLH4*, Mp*GRAS6/DELLA* and Mp*LOG*. Given their expression context it is likely that this group regulates certain aspects of thalloid totipotency/regeneration. Group 7 genes are Mp*SPL2* partners (Pearson > 0.8, coefficient average = 0.88) that overlap considerably with the Mp*ARF3* group, it also includes novel genes such as Mp*AThook1*, Mp*PIN3*, Mp*MIR11697* which targets Mp*YUC2* (Lin et al., 2016), Mp*PLINC* and Mp*GRAS12* (**Supplementary Figure S9J**). Group 8 (Pearson > 0.8 and coefficient average = 0.87) is defined by partners of Mp*NAC8* and includes 16 putative female enriched TFs (**Supplementary Figure S9K**) such as Mp*KNOX1*, Mp*BPC1*, Mp*TRIHELIX28* and *39*, Mp*PIN3*, and Mp*GRAS12.* Group 8 has overlaps with group 2/7 members such as Mp*SPL2*, Mp*ABI3B*, Mp*MIR11697* and it includes *MpR2R3-MYB1* a reproductive transition gene with highest expression in females (**Supplementary Figure S9K**).

Surprisingly, most auxin-related genes formed a large co-expression group (group 9) of 32 TFs (Pearson > 0.8, average coefficient = 0.87, **Figure 6**). Group 9 is defined by partners of the class A ARF, Mp*ARF1* (**Supplementary Figure S9L**), a TF regulating physiological and transcriptional auxin responses, gemmae development and gemmae dormancy (Kato et al., 2017). Additional members include the class B ARF, Mp*ARF2* (**Supplementary Figure S10**), which has been identified as a transcriptional repressor (Kato et al., 2015), the auxin signaling repressor Mp*IAA* (Flores-Sandoval et al., 2015; Kato et al., 2015), the NON-CANONICAL ARF Mp*NCARF* (Mutte et al., 2018), Mp*YUC2* an auxin biosynthetic gene (Eklund et al., 2015), Mp*PIN1* a putative intercellular auxin exporter (Bowman et al., 2017), Mp*AUX/LAX* an intercellular auxin importer (Bowman et al., 2017), Mp*C2HDZ* an auxin responsive gene (Mutte et al., 2018), and Mp*SHI* a putative YUC2 activator (Eklund et al., 2010), as well as additional TFs involved in sporeling patterning such as Mp*GRF* and Mp*ANT*. A cluster of bHLH genes including Mp*bHLH10, 42, 43, 45, 47*, and *48* are all members of this group. In agreement with recent works (Flores-Sandoval et al., 2018; Mutte et al., 2018) Mp*ARF3* is not co-expressed with any member of the auxin group except for Mp*NCARF* (Pearson = 0.78). A third set of putative auxin related genes showing independent co-expression dynamics includes the putative auxin receptor Mp*TIR1* as well as *NON-CANONICAL IAA* Mp*NCIAA*, which are associated with sporophytic genes (**Supplementary Figure S9D**). The dn-OPDA receptor in *Marchantia* Mp*COI* (Monte et al., 2018), is co-expressed with Mp*ARF1* (Pearson = 0.88). Mp*TAA* the major catalyst of Tryptophan into IPA, a precursor of auxin in *M. polymorpha* (Eklund et al., 2015) is also preferentially expressed in older sporophytes grouping with the sporophyte-specific MpYUC1 (Pearson = 0.91, **Figure 6** and **Supplementary Figure 9D**).

### Testing Co-expression Group Robustness in Thalloid Haploid Tissues

To examine co-expression group robustness and mitigate the biasing effects elicited by the non-thalloid sporelings, antheridia, and diploid sporophytic tissues, we generated a co-expression matrix without these three groups (**Supplementary Figure S11**). In this matrix, Mp*ARF1* forms a co-expression group with Mp*GRF*, Mp*MADS2*, Mp*COI*, and *MIR11707*, an amiR targeting Mp*AGO1* (Tsuzuki et al., 2015; Lin et al., 2016), that remains correlated with the auxin group. As expected, the auxin group now includes Mp*TAA* and Mp*NCIAA* but excludes Mp*NCARF* and Mp*AUX/LAX*. The biggest co-expression groups in this matrix are formed by reproductive genes or antheridiophore-specific genes (**Supplementary Figure S11**). Within the reproductive group, former groups 2 and 7 have merged to include Mp*ARF3*, Mp*SPL1-3*, Mp*C3HDZ*, Mp*CAMTA*, Mp*NCARF*, and Mp*TPL*, which was previously associated with antheridia. Mp*AUX/LAX* is grouped with the light signaling group, which has gained Mp*LUX*, formerly associated with sporophytes. Mp*WIP*, Mp*KAN*, and Mp*RR-A* form their own group, which has overlap with the auxin group.

### Co-expression Groups Are Dynamic at the Earliest Stages of Development

To examine how co-expression groups form at the earliest developmental transitions, we generated a matrix exclusive to sporeling RNA libraries. Multiple sporeling co-expression groups are formed that have different affinities with general co-expression groups (**Supplementary Figures S2**, **S3**, **S5**, **S12**). Auxin-related genes in particular show independent expression patterns prior to their clustering in later developmental stages. For example, the auxin signaling repressor Mp*IAA* forms a well-supported group (Pearson > 0.9) with genes that putatively promote cell proliferation such as the class I TCP gene Mp*TCP1* (Martín-Trillo and Cubas, 2010; Davière et al., 2014), and the day 1 sporeling-enriched TF Mp*GRF* (**Supplementary Figure S2**). Additional genes in this group include Mp*C4HDZ*, Mp*BBX3* and *5*, Mp*GRAS8*, Mp*DOF1* (Pearson > 0.9). Mp*GRAS4* is also correlated with GRF/TCP1, although more distantly (Pearson > 0.8). In sporelings, Mp*ARF1* and Mp*ARF2* form a well-supported group (Pearson = 0.93) with Mp*ARF1* more closely sharing expression with Mp*MIR11707/MIRAGO1* (Pearson = 0.99), similar to what is observed in the thallus. Mp*RAV*, Mp*bHLH48*, Mp*ANT*, Mp*R2R3-MYB17*, Mpb*HLH10, 21, 23* Mp*WIP* and Mp*LOG* are all co-expressed genes in this group (Pearson > 0.9). Mp*YUC2*, Mp*NCIAA*, and Mp*TIR* form separate groups from each other and independent from the Mp*ARF1/2* group. With Mp*TIR* and Mp*NCIAA* moderately close to each other (Pearson > 0.75). The largest co-expression group in sporelings consist of genes with high expression in 0-h sporelings. Genes included in this group are Mp*ARF3*, Mp*TAA*, Mp*PIN1*, Mp*SPL2*, Mp*WOX*, Mp*C3HDZ*, Mp*VAL* among others. Finally Mp*NCARF* is in another group clustering with Mp*HMGbox3*, Mp*RR-MYB6*, Mp*HD2/SAWADEE*, Mp*PIN4*, and Mp*IPT1* (Pearson > 0.9).

### The Auxin Co-expression Group Is Expressed Independently of the Single *Marchantia* Class C ARF and NCIAA

**FIGURE 7:**
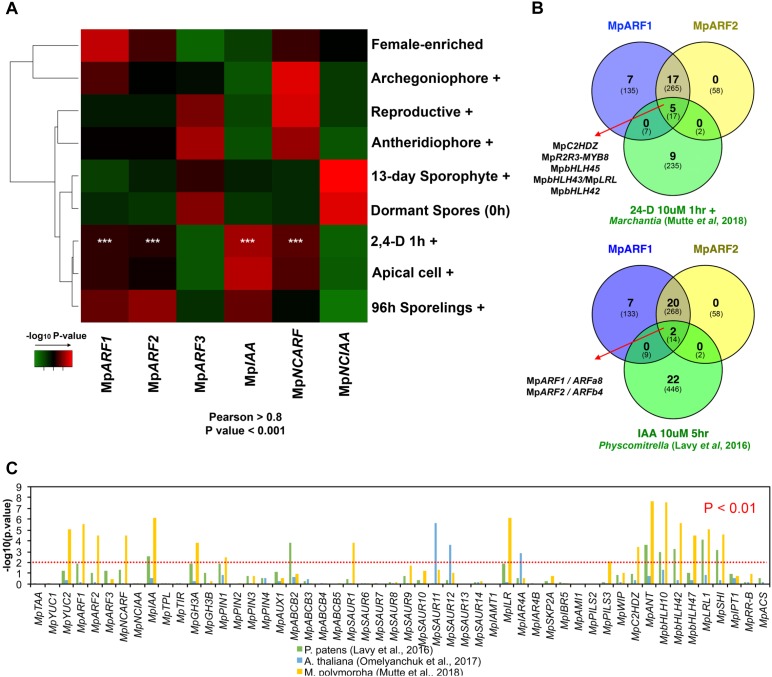
Auxin-related genes belong to a common co-expression network in *M. polymorpha* and other embryophytes. **(A)** Enrichment analysis heat map interrogating Mp*ARF1-3*, Mp*IAA*, Mp*NCARF*, and Mp*NCIAA* co-expression groups with tissue-enriched transcriptomes identified in our DGE analysis. High -log_10_ *P*-values are shown in red and are equivalent to highly supported enrichment *P*-values. For the comparison with the auxin induced transcriptome white asterisks indicate *P* < 0.01. **(B)** TFs shared between the Mp*ARF1* and Mp*ARF2* co-expression groups and auxin-induced TFs in *M. polymorpha* (above) and *P. patens* (below). The total number of shared genes (or orthologs) is indicated below. **(C)** Enrichment analysis of all putative auxin-related co-expression groups against auxin-induced genes in *M. polymorpha*, and uxin-induced orthologs in *P. patens* and *A. thaliana*.

To validate a putative auxin co-expression group we performed a co-expression analysis of most PB1-containing genes (Mp*ARF1-3*, Mp*IAA*, Mp*NCARF*, and Mp*NCIAA*) against all annotated *M. polymorpha* genes (**Figure 7**). Enrichment analysis (Fisher’s exact test) was used to compare co-expression groups (Pearson > 0.8 and *P* < 0.001) with the multiple datasets obtained from our DGE analysis (**Figure 2**, *P* < 0.01, logFC > 0). The Mp*ARF1*, Mp*ARF2*, Mp*IAA*, and Mp*NCAR*F co-expression groups were significantly composed of auxin-induced genes (Mutte et al., 2018) as well as apical cell-enriched transcripts (**Figure 7A**). Consistent with the TF co-expression matrix, the Mp*ARF3* and Mp*NCIAA* groups are not enriched in auxin-induced transcripts. TFs shared between the Mp*ARF1* and Mp*ARF2* groups and the Mutte et al. (2018) auxin-treated upregulated genes (**Figure 7B**) are Mp*C2HDZ*, Mp*R2R3-MYB8*, Mp*bHLH45*, Mp*bHLH43*/Mp*LRL*, and Mp*bHLH42*. The Mp*ARF1*, Mp*ARF2*, and Mp*IAA* groups were also enriched in 96-h sporeling transcripts, particularly the Mp*ARF2* group (**Figure 7A**). All groups had significant overlap with archegoniophore transcripts, except Mp*IAA*. Only the Mp*ARF3* and Mp*NCARF* groups were enriched with reproductive transition genes (**Figure 7A**). Finally, the Mp*NCIAA* group was significantly composed of dormant spores (**Supplementary Table S3**) and 13-day sporophyte enriched genes (**Figure 7A**). UpSet diagrams (**Supplementary Figure S13**) corroborated these observations with Mp*NCIAA* having the largest unique co-expression group (588 genes exclusive to NCIAA), followed by Mp*ARF3* (*N* = 138), Mp*NCARF* (*N* = 120), Mp*IAA* (*N* = 83), Mp*ARF1* (*N* = 58), and finally Mp*ARF2* (*N* = 14). The most correlated groups were Mp*ARF1*, Mp*ARF2*, Mp*NCARF*, and Mp*IAA* with 123 genes exclusively shared between them, followed by Mp*ARF1*, Mp*ARF2*, and Mp*IAA* (66 exclusively shared genes) and Mp*ARF1*, Mp*ARF2*, and Mp*NCARF* (51 exclusively shared genes). No gene was shared across all groups, with only 21 genes shared between Mp*ARF1-3*, Mp*NCARF* and Mp*IAA*. Finally, Mp*ARF3* and Mp*NCARF* co-expression groups shared exclusively 25 genes (**Figure 7A**).

Consistent with these results, the Mp*ARF1*, Mp*ARF2*, Mp*NCARF*, and Mp*IAA* groups share six GO-terms by biological process (**Supplementary Figure S14A**) and four GO-terms (biological process) are shared between Mp*ARF1*, Mp*ARF2* and Mp*IAA*. In contrast, the Mp*ARF3* group showed 12 specific GO-terms (**Supplementary Figure S14B**) while the Mp*NCIAA* group had 11 specific GO-terms by biological process (**Supplementary Figure S14C**).

### The Auxin Co-expression Group Is Conserved in Bryophytes

To further test the robustness of auxin-related co-expression groups, we compared them with differentially expressed genes (up and down-regulated) in auxin-treated *M. polymorpha* thalli (Mutte et al., 2018), *P. patens* protonemal tissue (Lavy et al., 2016) and *Arabidopsis* root tissue (Omelyanchuk et al., 2017) using enrichment analysis. The co-expression groups of Mp*ARF1*, Mp*ARF2*, Mp*NCARF*, Mp*IAA*, Mp*YUC2*, Mp*GH3A*, Mp*PIN1*, Mp*SAUR1*, Mp*ILR1*, Mp*PILS3*, Mp*C2HDZ*, Mp*ANT*, Mp*bHLH10*, Mp*bHLH42*, Mp*bHLH47*, Mp*bHLH43*/Mp*LRL*, and Mp*SHI* showed significant similarity (*P* < 0.01, Fisher’s exact-test) with DEGs in auxin-treated *Marchantia* plants (**Figure 7C**). Meanwhile, the Mp*IAA*, Mp*ABCB2*, Mp*ANT*, Mp*bHLH10*, Mp*bHLH42*, Mp*bHLH47*, Mp*bHLH43*/Mp*LRL*, and Mp*SHI* groups showed significant resemblance (*P* < 0.01) to *Physcomitrella* auxin treated upregulation profiles, with Mp*ARF1*, *PIN1*, Mp*GH3A*, and Mp*ILR* groups overlapping at *P* < 0.05 (**Figure 7C**). In contrast, only the Mp*SAUR11*, Mp*SAUR12*, and Mp*IARF4A* groups showed significant enrichment with auxin-treated *Arabidopsis* plants orthologs. This suggests a conservation of auxin co-expression groups across bryophytes, although the only TFs shared between the Mp*ARF1* and Mp*ARF2* co-expression groups and the moss auxin-induced transcriptome are the class A and B ARF orthologs themselves, ARFa8 and ARFb4 (**Figure 7B**).

### Analysis of Mp*ARF3* Gain and Loss-of-Function Mutant Transcriptomes Confirms Validity of Co-expression Groups

**FIGURE 8:**
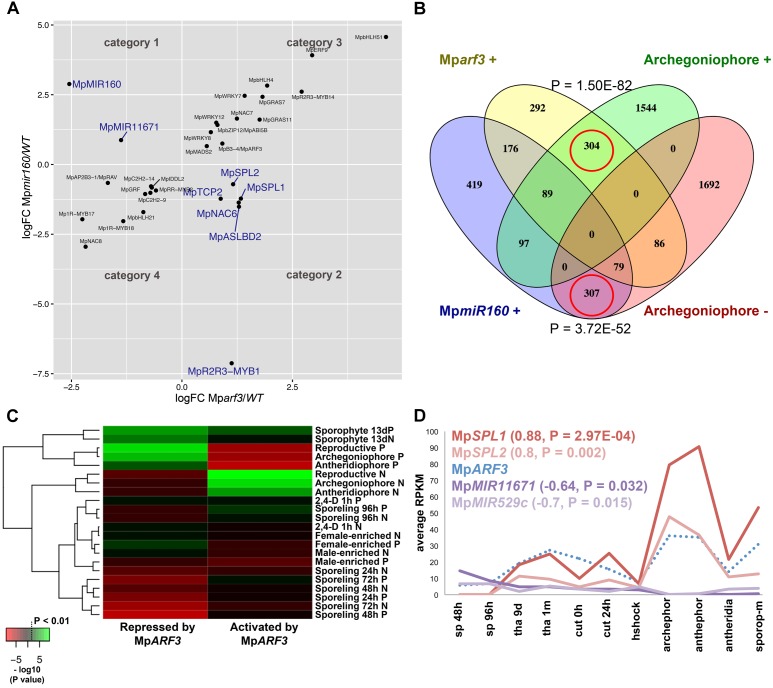
The Mp*ARF3*/Mp*MIR160* module controls reproductive transitions in *M. polymorpha.* **(A)** Differentially expressed TFs in Mp*arf3* and Mp*mir160* transcriptomes fall into four categories depending on logFC between wild-type and mutant reads. Results are shown for the edgeR pipeline with a *P* < 0.01 cut-off. Genes in blue are consistent with Mp*miR160*-dependent repression of Mp*ARF3*. **(B)** Venn diagram showing overlap of genes upregulated in Mp*arf3* and Mp*mir160* transcriptomes with genes whose expression is enriched or downregulated in archegoniophores. **(C)** Enrichment analysis (Fisher’s exact test) of genes activated (category 1) or repressed (category 2) by Mp*ARF3* against all differentially expressed genes obtained from previous analyses in **Figure 2**. Significant enrichment values are shown in green. **(D)** Expression dynamics of Mp*ARF3*, Mp*SPL1*, Mp*SPL2*, Mp*MIR11671*, and Mp*MIR529c* using average RPKM values. Pearson coefficients and associated *P*-values are shown in reference to Mp*ARF3*.

We performed DGE analysis (edgeR and DESeq2) in previously isolated Mp*arf3*/M*pmir160* CRISPR mutants (Flores-Sandoval et al., 2018) to test whether: (1) loss of Mp*ARF3* disrupts the auxin co-expression group, (2) genes co-expressed with Mp*ARF3* are altered in mutant alleles, and (3) Mp*arf3* or Mp*mir160* transcriptomes could facilitate identification of genes responsible for developmental aspects of their respective mutant phenotypes. The edgeR analysis identified 2874 differentially expressed genes in Mp*arf3* compared to the wild type (*P* < 0.01, 1524 upregulated). Similarly, 2864 differentially expressed genes were identified in comparisons of Mp*miR160* mutants with the wild type (*P* < 0.01, 1572 upregulated). To filter out steady-state false positives and highlight genes dependent on Mp*ARF3* activity, we searched for genes with converse behaviors in the Mp*arf3* and Mp*mir160* transcriptomes. Genes down-regulated in Mp*arf3* and upregulated in Mp*miR160* mutants were considered to be activated, either directly or indirectly, by Mp*ARF3* (Category 1, *N* = 112; **Supplementary Table S18**). Conversely, genes upregulated in Mp*arf3* and down-regulated in Mp*mir160* mutants were considered repressed, either directly or indirectly, by Mp*ARF3* (Category 2, *N* = 115; **Supplementary Table S19**). Strikingly, genes with similar dynamics, i.e., upregulated (Category 3, *N* = 501; **Supplementary Table S20**) or down-regulated (Category 4, *N* = 470; **Supplementary Table S21**) in both mutants were ∼4× more abundant, suggesting a large cohort of genes affected by secondary feedback effects independent of the Mp*miR160*/Mp*ARF3* regulatory module (**Figure 8A**). The most prevalent protein family found in Category 1 was peroxidases (PF00141, **Supplementary Table S22**), while Category 2 had an abundance of FB_lectins (PF07367, **Supplementary Table S23**), four of which were in tandem arrays in scaffold 118 and with high resemblance to fungal lectin genes (Bowman et al., 2017).

Although no TFs were identified as activated by Mp*ARF3* (Category 1), two miR precursors fell into this category, with Mp*MIR160* (Mapoly0002s0211) and Mp*MIR11671* (Mapoly0239s0004) supported by both edgeR and DEseq2 analyses (**Figure 8A**). Meanwhile, a third miR precursor, Mp*MIR529c* (Mapoly0006s0020) was also identified as activated by Mp*ARF3* using DESeq2 (**Supplementary Figure S14D**). We had previously suggested that Mp*ARF3* forms a negative feedback loop with Mp*miR160* to control both developmental transitions and differentiation vs. totipotency in a context-dependent fashion (Flores-Sandoval et al., 2018).

The other two miRs identified in our analysis target *SPL* transcripts (Tsuzuki et al., 2015; Lin et al., 2016), with Mp*miR11671* targeting Mp*SPL1* (Mapoly0014s0224, orthologous to SPL8 of *Arabidopsis*) and Mp*miR529c* targeting Mp*SPL2* (Mapoly0014s0223, orthologous to SPL3-5 of *Arabidopsis*). Consistent with these results, both Mp*SPL1* and Mp*SPL2* appeared as category 2 genes (i.e., repressed by Mp*ARF3*) using both edgeR and DEseq2, and supported by protein family GO-terms (*P* = 0.0006924). Additional TFs identified as downregulated by Mp*ARF3* included Mp*TCP2* (Mapoly0001s0298), a FEZ-like NAC domain ortholog Mp*NAC6* (Mapoly0175s0015), the class Ia ASL1 gene Mp*ASLBD2* (Mapoly0008s0060), and Mp*R2R3-MYB1* (Mapoly0001s0061). Mp*R2R3-MYB1* was most dramatically downregulated in Mp*mir160* alleles (logFC = -7, **Figure 8A**).

**FIGURE 9:**
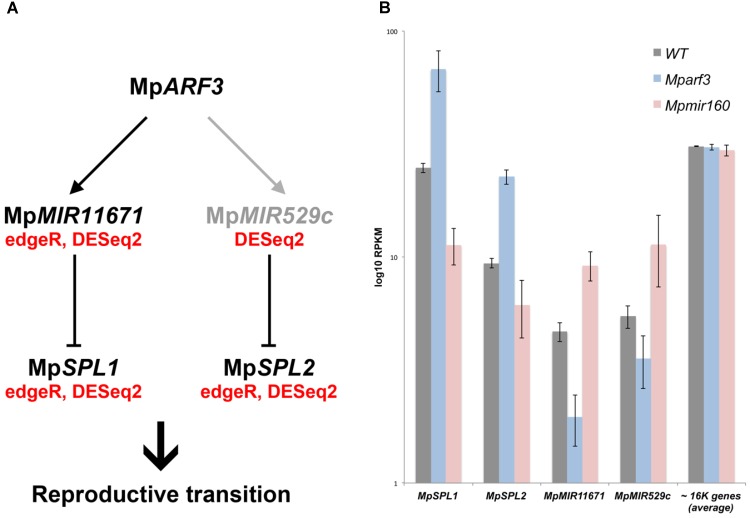
Model for Mp*ARF3* dependent regulation of reproductive phase changes in *M. polymorpha*. **(A)** Putative regulatory relationships between Mp*ARF3* and Mp*SPL1-2* as supported by RNA-Seq data. Relationships supported by two (edgeR and DESeq2) or one statistical analyses (DESeq2) are indicated. Reproductive phase change roles are attributed to *M. polymorpha* SPL genes given their common enrichment in gametangiophores. **(B)** Average log_10_RPKM values (biological triplicates) for Mp*SPL1*, Mp*SPL2*, Mp*MIR11671*, and Mp*MIR529c* in our experimental conditions. The average RPKM value for all mapped genes (*N* = 16520) per library is ∼30. Error bars show SD.

Three of the downregulated TFs (Mp*SPL1, 2* and Mp*R2R3-MYB1*) as well as Mp*ARF3* itself were classified as reproductive genes in our wild-type DGE analysis (**Figure 5**). Furthermore, we had previously characterized Mp*ARF3* as an antagonist of reproductive transitions, with Mp*mir160* alleles insensitive to gamentangiophore-inducing far-red light treatment and Mp*arf3* alleles as hypersensitive to far-red light treatment (forming more gametangiophores per area than wild type; Flores-Sandoval et al., 2018). We therefore measured the overlap between genes enriched in gametangiophores (either male or female) and the Mp*arf3* or Mp*miR160* mutants. A significant number of genes (*N* = 304, *P* = 1.5 × 10^-82^, Hypergeometric test) were upregulated in both Mp*arf3* mutants and wild-type archegoniophores (**Figure 8B**). Consistently, a significant number of genes were upregulated in Mp*miR160* mutants and downregulated in archegoniophores (*N* = 307, *P* = 3.7 × 10^-52^, Hypergeometric test). Enrichment analysis (Fisher’s exact test) using the datasets obtained in our DGE analysis (edgeR, *P* < 0.01) show that overall transcripts activated by Mp*ARF3* (category 1) are similar to those downregulated in wild type gametangiophores (*P* < 0.01, **Figure 8C**). Conversely, transcripts repressed by Mp*ARF3* (category 2) are significantly upregulated in wild type gametangiophores, in particular the subset of reproductive transition genes and not the female or male-enriched genes (**Figure 8C**). Additionally, 13-day sporophytes also have transcripts that seem to be controlled by Mp*ARF3* although with an ambiguous trend unlike that observed for reproductive tissues. Although some auxin-related or auxin-induced genes were differentially expressed in Mp*arf3* transcriptomes (Flores-Sandoval et al., 2018), not a single auxin-induced gene had converse expression patterns in the Mp*arf3* and Mp*mir160* transcriptomes (**Figure 8A**). Finally, the expression dynamics of the candidate TF/MIR pairs shows that Mp*ARF3*, Mp*SPL1* and *2* expression patterns are positively correlated with peaks of expression in gametangiophores (**Figure 8D**), while the Mp*MIR11671* and Mp*MIR529c* precursors are negatively correlated with Mp*ARF3* and show down-regulation in gametangiophores. Thus, an inverse relationship exists between Mp*ARF3* activity and expression of the Mp*SPL1-2*/Mp*MIR529c*/Mp*MIR11671* modules in *M. polymorpha* (**Figures 8D**, **9B**).

## Discussion

This study provides a first attempt at characterizing DGE patterns and co-expression groups throughout the life cycle of *M. polymorpha* (**Figure 1**). Given their hierarchal regulatory character, TFs were chosen as a proxy for the thousands of genes differentially expressed between cell and tissue types. DGE analysis reveals that only a minority of TFs show dramatic fold-changes between developmental transitions in *M. polymorpha*. However, this figure is qualified by the limited number of tissues and environmental conditions sampled in our analysis (**Figure 2**). These differentially expressed TFs are candidates for functional analysis to assess whether they determine cell identity or broader processes required for tissue patterning, i.e., meiosis, cell division, expansion, stress-responses.

The relatively small number of annotated TFs (∼400) in *M. polymorpha* (Bowman et al., 2017) facilitated construction of a co-expression matrix using 11 tissues, including sporelings, thallus, gametangiophores, antheridia and sporophytes. This matrix resolved multiple discrete groups with variable levels of overlap and putative interactions (**Figure 6**). As expected, some groups correspond to tissue-specific TF enrichment (e.g., sporophytes, antheridia, archegoniophores or regenerating tissue). Surprisingly, a second kind of group that did not show enrichment in any one type of library (**Supplementary Figures S9E,G,I,L**) was also identified. These groups include the partners of Mp*ARF1* or Mp*TOC1*, which are involved in auxin and light/circadian responses, respectively. Members of these co-expression groups were differentially expressed in multiple stages but did not change dramatically across our pair-wise comparisons, likely due to lack of inductive environmental conditions or because they are under tight regulatory feedbacks. Thus, our data suggests developmental transitions are also defined by the additive influence of multiple members of a co-expression group.

### TF Functions Predicted by Our Analysis

#### Totipotency

A set of TFs that could represent regulating factors controlling aspects of totipotency include the *LAS* ortholog Mp*GRAS4, MpANT*, and *MpARF2.* Mp*GRAS4* is enriched in the apical cell and both 24 and 48 h sporelings. It is further nested in a co-expression group enriched in 24-h regenerating tissue following wounding (**Supplementary Figure S9F**). Thus, Mp*GRAS4* is expressed in tissues united by active cell proliferation. Second, the *ANT/PLT/BBM* ortholog, Mp*ANT*, is enriched exclusively in apical cells and 96 h sporelings, raising the possibility that its action is downstream of Mp*GRAS4.* Mp*ANT* is also co-expressed in the auxin cluster (**Supplementary Figure S10**), suggesting that it may be auxin-inducible, as occurs in angiosperm model systems (Galinha et al., 2007; Ding and Friml, 2010). Consistent with this scenario, Mp*ARF2* is also an apical-cell enriched gene whose expression steadily increases from 72-h sporelings (in parallel with Mp*ARF1*, **Supplementary Figure S4A**). It is plausible, given that Mp*ARF1* and Mp*ARF2* act as respective activators or repressors of transcription (Kato et al., 2015), that antagonism between Mp*ARF1* and Mp*ARF2* at 72 h stabilizes Mp*ANT* expression in 96 h sporelings, subsequently forming a co-expression group throughout development. Given that *LAS* has key roles specifying axillary meristems in angiosperms, and that *ANT/PLT/BBM* orthologs act in meristems in both angiosperms (Aida et al., 2004) and mosses (Aoyama et al., 2012), there might be more overlap between haploid and diploid meristems than previously acknowledged (Frank and Scanlon, 2015).

#### Cell Proliferation and Auxin

Additional genes may play a role in cellular processes that contribute to, but do not specify, meristematic tissues. For example, a cluster represented by the 24-h sporelings involving Mp*TCP1*, Mp*GRF*, Mp*C4HDZ* and Mp*IAA* genes could represent a cell proliferation group. Mp*GRF* in particular is highly expressed in the first day of sporeling germination, but it is also co-expressed with auxin-related genes throughout the life cycle (**Figure 6A** and **Supplementary Figure S11**). Auxin signaling in 24-h sporelings may be constrained by the presence of the known Mp*IAA* auxin signaling repressor (Flores-Sandoval et al., 2015; Kato et al., 2015). Given that key aspects of auxin physiology in *M. polymorpha* involve organ differentiation (Eklund et al., 2015; Flores-Sandoval et al., 2015; Kato et al., 2015), it is tempting to speculate that there may be an antagonism between auxin-dependent differentiation and cell proliferation elicited by Mp*GRF* and other TFs in *M. polymorpha*.

#### Differentiation Factors

An auxin-inducible gene (Mutte et al., 2018) characterized in *M. polymorpha* is the IDDL-like Zinc Finger Mp*WIP* TF, which is essential for air pore differentiation (Jones and Dolan, 2017). Consistently, Mp*WIP* is upregulated in 96-h sporelings at the time of photosynthetic tissue proliferation (**Supplementary Figure S5**). Mp*WIP* is co-expressed (**Supplementary Figure S9H**) with the GARP TF Mp*KAN*, whose orthologs influence organ polarity in angiosperms (Eshed et al., 2001). Mp*KAN* and its co-expression partner Mp*WRKY8* are in turn clearly enriched in 0-h cut thalli that lack apical notches and haven’t initiated regeneration. One hypothesis is that members of this group may regulate key aspects of differentiation possibly downstream of auxin and light signaling, given their overlap in expression patterns (Mp*KAN* and Mp*ARF1* have a Pearson coefficient of 0.76, while Mp*PIF* and Mp*KAN* have a coefficient of 0.8).

#### Reproductive Genes in *Marchantia*

Comparisons between gametangiophore datasets facilitated distinctions between reproductive transition TFs (enriched in both sexes) vs. female or male-enriched TFs. Mp*BNB* served as a marker to validate this group given its role in promoting formation of gametangiophores irrespective of sex (Yamaoka et al., 2018). A co-expression group including Mp*SPL1*, Mp*SPL2*, Mp*C3HDZIP*, Mp*ABI3*B, Mp*ARF3*, among others correlates with the vegetative to reproductive transition and is supported by mutant transcriptomes (see below section). Interestingly, auxin-repressed genes are significantly represented in archegoniophore and antheridiophore-enriched transcripts (**Supplementary Figure S4B**), suggesting a repressor(s) of auxin signaling is a member of the reproductive transition group. Male-enriched TFs were refined by the presence of antheridia libraries, which suggest a robust set of male gamete-enriched TFs and these have been extensively described in previous studies (Higo et al., 2016). Mp*RWP2*/Mp*MID* could provide an interesting candidate for an antheridia specific factor given that its orthologs specify *minus* gametes in green algae (Ferris and Goodenough, 1997).

#### Extended Parental to Zygotic Transition in *M. polymorpha*

The identification of female and antheridia-enriched TFs allowed comparisons with upregulated TFs in 13-day sporophytes, consisting of ∼10 cells and at which point sporogenous tissue differentiation has not commenced (Frank and Scanlon, 2015). Female-enriched TFs shared with young sporophytes include members of 10 TF families. Male-enriched TFs shared with young sporophytes involve members of 7 TF families, although Mp*BELL4* is the only identified TF exclusively upregulated in antheridia and young sporophytes (**Figure 2B**). Enrichment analysis (**Supplementary Figure S6**) suggests that only archegoniophore-enriched genes are significantly represented in 13-day sporophyte transcriptomes. Thus, the possibility of RNA contamination from maternal tissues still remains as a plausible explanation to this phenomenon.

### Class C ARF and Mp*NCIAA* Genes Are Independent of Other Auxin Related Genes

The discovery of a putative auxin co-expression group among transcription factors (**Figure 6**) coincides with a similar PIN1-related co-expression group observed in moss (Ruprecht et al., 2017b), suggesting an ancestral origin for the auxin co-expression network in land plants. The robustness of the auxin TF co-expression group was tested using whole-genome co-expression analysis for candidate genes and comparisons with the only available auxin-inducible transcriptome in *M. polymorpha* (Mutte et al., 2018). Enrichment analysis supports significant overlap between Mp*ARF1*, Mp*ARF2*, Mp*IAA*, Mp*NCARF*, Mp*YUC2*, Mp*SAUR1*, Mp*PIN1*, Mp*GH3A*, and Mp*ILR1* co-expression groups and the *M. polymorpha* auxin-upregulated transcriptome (**Figures 7A,C**). Other TFs within the auxin co-expression group but strictly not involved in auxin biosynthesis, transport, conjugation or responses include Mp*ANT*, Mp*SHI*, Mp*bHLH10*, *43, 45* and *47*, Mp*MIR11707*, *MpR2R3-MYB8*, and Mp*C2HDZ* (**Figure 7C** and **Supplementary Figure S12**). They could represent upstream or downstream elements regulating the module (Mutte et al., 2018) and are suitable candidates for future characterization. Importantly, many of these genes are also part of the auxin-inducible transcriptome of *Physcomitrella* but not *Arabidopsis* (**Figure 7C**), suggesting they were key components of the auxin co-expression network in the ancient bryophyte ancestor. As Mp*bHLH43/LRL* possibly mediates ectopic rhizoid formation (Breuninger et al., 2016) in response to exogenous auxin in *M. polymorpha* its association was anticipated. Furthermore, 5 of the 14 TFs induced by auxin (**Supplementary Table S24**) in *M. polymorpha* are found within the Mp*ARF1* and Mp*ARF2* co-expression groups (**Figure 7B**). Notably, the class C ARF, Mp*ARF3*, a gene that is not necessary for physiological and transcriptional auxin responses (Flores-Sandoval et al., 2018; Mutte et al., 2018), forms part of an independent co-expression group highly active in reproductive structures (**Figure 8** and **Supplementary Figure S9**). However, Mp*ARF3* still shares members with Mp*NCARF* (72 genes), Mp*ARF1*/Mp*ARF2*/Mp*NCARF/MpIAA* (21 genes) and Mp*NCIAA* (9 genes, **Supplementary Figure S13**) co-expression groups. The significance of this connectivity was tested (**Supplementary Table S25**), providing ways in which Mp*ARF3* competes for Mp*ARF1* and Mp*ARF2* targets in an auxin-independent manner. Importantly, the Mp*ARF3* and Mp*NCARF* co-expression groups are enriched in reproductive transition genes (**Figure 7A**), opening the possibility of interaction at this developmental stage. Mp*NCIAA* is another PBI-containing factor that forms an independent group with sporophyte-enriched transcripts (**Figure 7** and **Supplementary Figure S9**) and is also not essential for transcriptional auxin responses (Mutte et al., 2018). Consistent with their auxin independence, both class C ARFs and NCIAAs evolved before the origin the canonical auxin-signaling pathway (and land plants), and thus they may act in independent regulatory networks. The putative roles of *NCIAA* genes in patterning sporophytic development are of interest although these genes are also expressed in haploid tissues in *M. polymorpha*.

### RNA-Seq Data Supports the Role of Mp*ARF3* as an Inhibitor of Reproductive Transitions

We have previously described Mp*ARF3* as a repressor of differentiation in multiple developmental contexts. For example, Mp*arf3* mutants display developmental transition, defects, such as ectopic differentiation of air pores, scales, pegged rhizoids, and gametophores, and are deficient in forming undifferentiated gemmalings (Flores-Sandoval et al., 2018; Mutte et al., 2018). Meanwhile strong Mp*ARF3* gain-of-function alleles form undifferentiated calli whose cell identity resembles that of young sporelings or prothalli (Flores-Sandoval et al., 2018). Thus, we anticipated the transcriptome data should reflect aspects of these developmental defects. Using gain- and loss-of-function Mp*ARF3* transcriptomes, were able to discern secondary feedback, treatment-dependent and steady state effects and thus assign with more confidence genes genuinely dependent on the Mp*MIR160*/Mp*ARF3* regulatory module (category 1 and 2 genes, **Figure 8**). Indeed, enrichment analysis demonstrated that Mp*ARF3* represses, directly or indirectly, genes that promote reproductive transitions (**Figure 8**). Consistently, Mp*ARF3* activates genes that inhibit production of, or at least are downregulated in, sexual organs. We therefore propose the hypothesis (**Figure 9A**) that Mp*ARF3* inhibits the reproductive transition in *M. polymorpha* via activation of the Mp*MIR11671* and Mp*MIR529c* precursors, which in turn target Mp*SPL1* and MpS*PL2*. This appears plausible, given the observed Mp*SPL1* and Mp*SPL2* RPKM values in Mp*arf3/mir160* mutant backgrounds (**Figure 9B**) and the roles of SPL genes in regulating heteroblastic changes and specifically in promoting reproductive transitions in angiosperms (Huijser and Schmid, 2011) and possibly mosses (Cho et al., 2012).

## Author Contributions

EF-S generated data for **Figures 1–5**. EF-S and FR generated data for **Figures 6–8**. FR created scripts for enrichment analysis and GO-terms pipelines. EF-S designed all figures, except **Figures 6**, **7**, which were jointly designed by EF-S and FR. EF-S and JB co-wrote the manuscript and interpreted the data. All authors analyzed and revised the data.

## Conflict of Interest Statement

The authors declare that the research was conducted in the absence of any commercial or financial relationships that could be construed as a potential conflict of interest.

## Footnotes

1 https://phytozome.jgi.doe.gov/

2 https://pfam.xfam.org/

## Supplementary Material

FIGURE S1 **(A)** Frequency of log2FC values in pairwise comparisons performed in this study. **(B)** UpSet diagram of upregulated genes (logFC), including all curated TFs, in this study. Red asterisk indicates TFs not differentially expressed in any DGE analysis comparison.

FIGURE S2 Identification of co-expression groups of 24-h enriched TFs in *M. polymorpha* sporelings. RPKM values are shown for all replicates from 0 to 96 h.

FIGURE S3 Identification of co-expression groups of 48-h enriched TFs *M. polymorpha* sporelings. RPKM values are shown for all replicates from 0 to 96 h.

FIGURE S4 Auxin-signaling and biosynthetic gene expression during sporeling development. **(A)** RPKM averages of Mp*ARF1*, Mp*ARF2*, Mp*ARF3*, Mp*IAA*, Mp*TAA*, and Mp*YUC2* during the first four days of sporeling development. Error bars indicate SD. Upregulation of Mp*ARF1* and Mp*ARF2* and downregulation of Mp*IAA* at 96 h are statistically supported. *P*-values marked are from edgeR analysis. **(B)** Enrichment analysis of auxin upregulated and downregulated genes probed against multiple developmental RNA-Seq libraries obtained from DGE analysis. The tissue with the highest resemblance to the auxin-upregulated transcriptome is 96-h sporelings.

FIGURE S5 Identification of co-expression groups of 72 and 96-h enriched TFs *M. polymorpha* sporelings. RPKM values are shown for all replicates from 0 to 96 h.

FIGURE S6 Sporophytic gene expression in *M. polymorpha.* **(A)** Enrichment analysis of 13-day and JGI manually dissected sporophytes shows significant enrichment between the transcriptomes. Genes classified as female-enriched (**Supplementary Table S10**) are also statistically represented in 13-day sporophyte transcriptomes. **(B)** Venn Diagram of shared TFs between antheridia, archegoniophore, 13-day and JGI sporophytes. Putative maternal, paternal and common TFs continuing expression in 13-day sporophytes are annotated. The + symbol indicates upregulation (logFC > 0).

FIGURE S7 UpSet diagram of tissue-specific TF enrichment at logFC > 1. Red asterisk shows eight uniquely upregulated TFs in apical cells at logFC > 1 compared to other tissues.

FIGURE S8 Distance dendrogram generated with hclust (R Version 2.3.2) of all annotated *M. polymorpha* TFs (**Supplementary Table S1**) in 11 tissue libraries using RPKM > 1 as a threshold.

FIGURE S9 RPKM dynamics of TF/response/biosynthetic genes obtained in our analysis. Pearson coefficients above 0.8 were used to define the Mp*BELL4* **(A)**, Mp*ARF3* **(B)**, Mp*BELL1* **(C)**, Mp*NCIAA* **(D)**, Mp*bHLH14/RSL* **(E)**, Mp*GRAS4* **(F)**, Mp*TOC1* **(G)**, Mp*GARP1/KAN* **(H)**, Mp*GARP8/GLD* **(I)**, Mp*SPL2* **(J)**, Mp*NAC8* **(K)**, and Mp*ARF1* **(L)** coxpression groups. Accompanying TFs are indicated at the left of each graph.

FIGURE S10 RPKM dynamics of the putative auxin co-expression group genes.

FIGURE S11 **(A)** Heatmap of Pearson coefficient matrix of all annotated *M. polymorpha* TFs, MIR precursors and putative hormonal genes (**Supplementary Table S16**) RPKM values above 0.75 (to include Mp*ANT*) in sporeling tissues. Putative groups were delimited by sets of genes sharing coefficients above 0.8. Genes putatively involved in auxin biosynthesis, perception, signaling and transport are mapped, together with accompanying partners in smaller fonts. **(B)** Distance dendrogram of tissue libraries used in the analysis. Average replicate RPKM values were used per library. Scale for heatmap indicates Pearson coefficients.

FIGURE S12 **(A)** Heatmap of Pearson coefficient matrix of all annotated *M. polymorpha* TFs, MIR precursors and putative hormonal genes with RPMK values above 5 (**Supplementary Table S17**) in thalli and gametangiophores. Putative groups were delimited by sets of genes sharing coefficients above 0.8. Genes putatively involved in auxin biosynthesis, perception, signaling and transport are mapped, together with accompanying partners in smaller fonts. **(B)** Distance dendrogram of tissue libraries used in the analysis. Average replicate RPKM values were used per library. Scale for heatmap indicates Pearson coefficients.

FIGURE S13 **(A)** UpSet diagram showing shared co-expressed genes (cut-offs indicated) between most PBI-containing genes in *M. polymorpha*. Numbers of genes exclusive or shared between groups are shown. Co-expression analyses were performed for all expressed genes in our RPKM matrix.

FIGURE S14 **(A)** Significant GO-terms (Biological Process) found in the Mp*ARF1*, Mp*ARF2*, Mp*NCARF*, and Mp*IAA* co-expression groups. X-axis indicates *P*-Values. **(B)** Significant GO-terms (Biological Process) found in the Mp*ARF3* co-expression group. X-axis indicates *P*-values. **(C)** Significant GO-terms (Biological Process) found in the Mp*NCIAA* co-expresion group. X-axis indicates *P*-values. **(D)** TFs differentially expressed in Mp*arf3* and Mp*miR160* transcriptomes using DESeq2 (*P* < 0.01 cut-off). Genes in blue are consistent with Mp*miR160*-dependent repression of Mp*ARF3*.

TABLE S1 TFs, MIRs and hormonal genes and accession numbers used in this paper.

TABLE S2 Shared upregulated genes between DEG analysis comparisons at logFC > 0.

TABLE S3 Differentially expressed TFs and genes in 24-h sporelings using edgeR.

TABLE S4 Differentially expressed TFs and genes in 48-h sporelings using edgeR.

TABLE S5 Differentially expressed TFs and genes in 72-h sporelings using edgeR.

TABLE S6 Differentially expressed TFs and genes in 96-h sporelings using edgeR.

TABLE S7 Differentially expressed TFs and genes in archegoniophores using edgeR.

TABLE S8 Differentially expressed TFs and genes in antheridiophores using edgeR.

TABLE S9 Differentially expressed TFs and genes shared in archegoniophores and antheridiophores (reproductive TFs) using edgeR.

TABLE S10 Archegoniophore-enriched TFs and genes using edgeR.

TABLE S11 Antheridiophore-enriched TFs and genes using edgeR.

TABLE S12 Differentially expressed TFs and genes in antheridia using edgeR.

TABLE S13 Differentially expressed TFs and genes in 13-day sporophytes using edgeR.

TABLE S14 Shared upregulated genes between DEG analysis comparisons at logFC > 1.

TABLE S15 Pearson coefficient matrix of TFs for **Figure 6**.

TABLE S16 Pearson coefficient matrix of TFs for **Supplementary Figure S11**.

TABLE S17 Pearson coefficient matrix of TFs for **Supplementary Figure S12**.

TABLE S18 Category 1 genes from Mparf3 (x) and Mpmir160 (y)transcriptomes.

TABLE S19 Category 2 genes from Mparf3 (x) and Mpmir160 (y)transcriptomes.

TABLE S20 Category 3 genes from Mparf3 (x) and Mpmir160 (y)transcriptomes.

TABLE S21 Category 4 genes from Mparf3 (x) and Mpmir160 (y) transcriptomes.

TABLE S22 Go analysis (PF-terms) of Category 1 genes from Mparf3 (x) and Mpmir160 (y) transcriptomes.

TABLE S23 Go analysis (PF-terms) of Category 2 genes from Mparf3 (x) and Mpmir160 (y) transcriptomes.

TABLE S24 Differentially expressed TFs in auxin-induced tissue (1 h) from Mutte et al. (2018) using edgeR.

TABLE S25 Enrichment-analysis between Mp*ARF1-3*, Mp*IAA*, Mp*NCARF*, and Mp*NCIAA* co-expression groups, *P*-values are shown.
