# Supplementary figures and images for: Co-expression and Transcriptome Analysis of Marchantia polymorpha Transcription Factors Supports Class C ARFs as Independent Actors of an Ancient Auxin Regulatory Module
Source: Front Plant Sci. 2018 Oct 1;9:1345. doi: 10.3389/fpls.2018.01345 (PMC6174852; doi:10.3389/fpls.2018.01345)

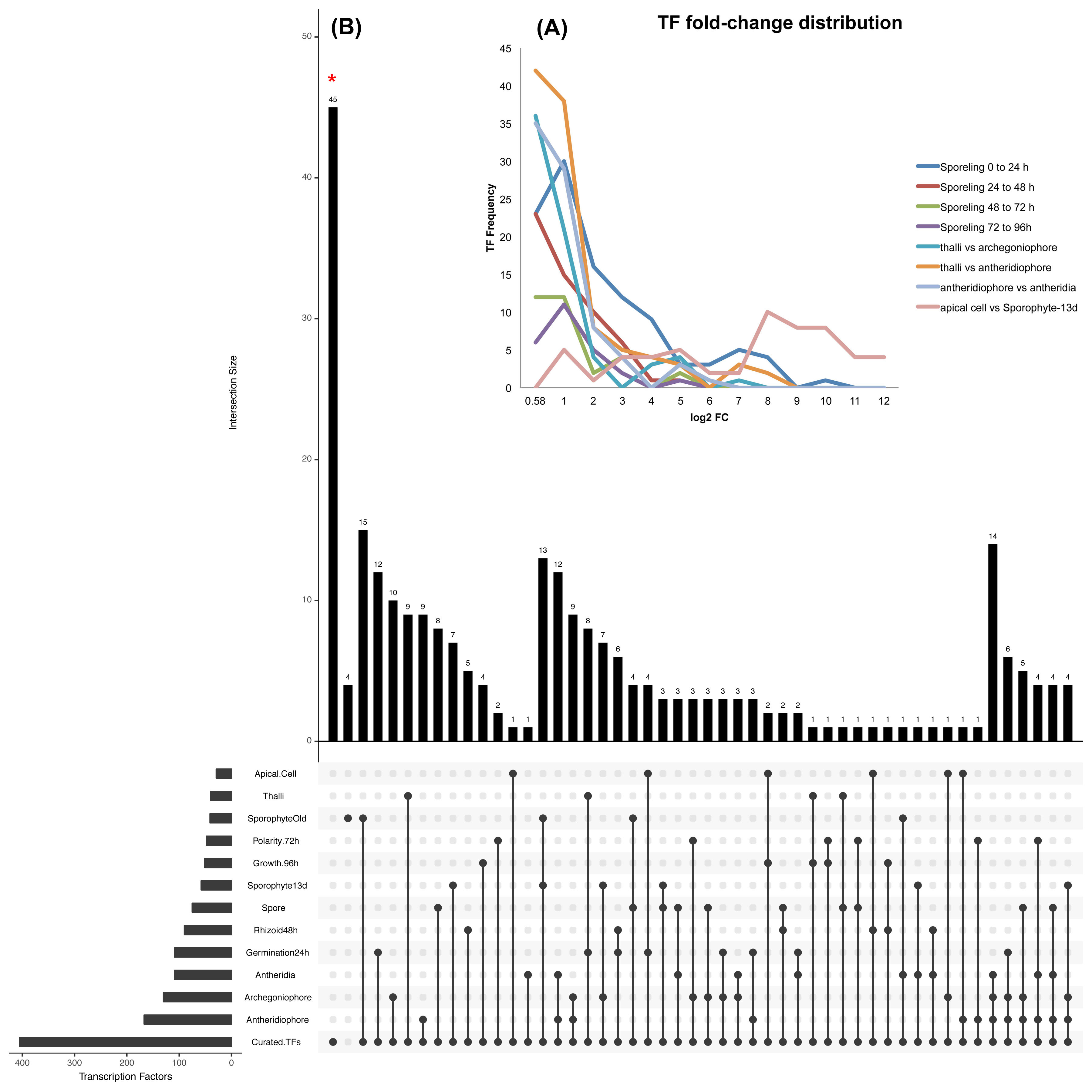

Supplement: FIGURE S1 — (A) Frequency of log2FC values in pairwise comparisons performed in this study. (B) UpSet diagram of upregulated genes (logFC), including all curated TFs, in this study. Red asterisk indicates TFs not differentially expressed in any DGE analysis comparison. [file Image_1.JPEG]

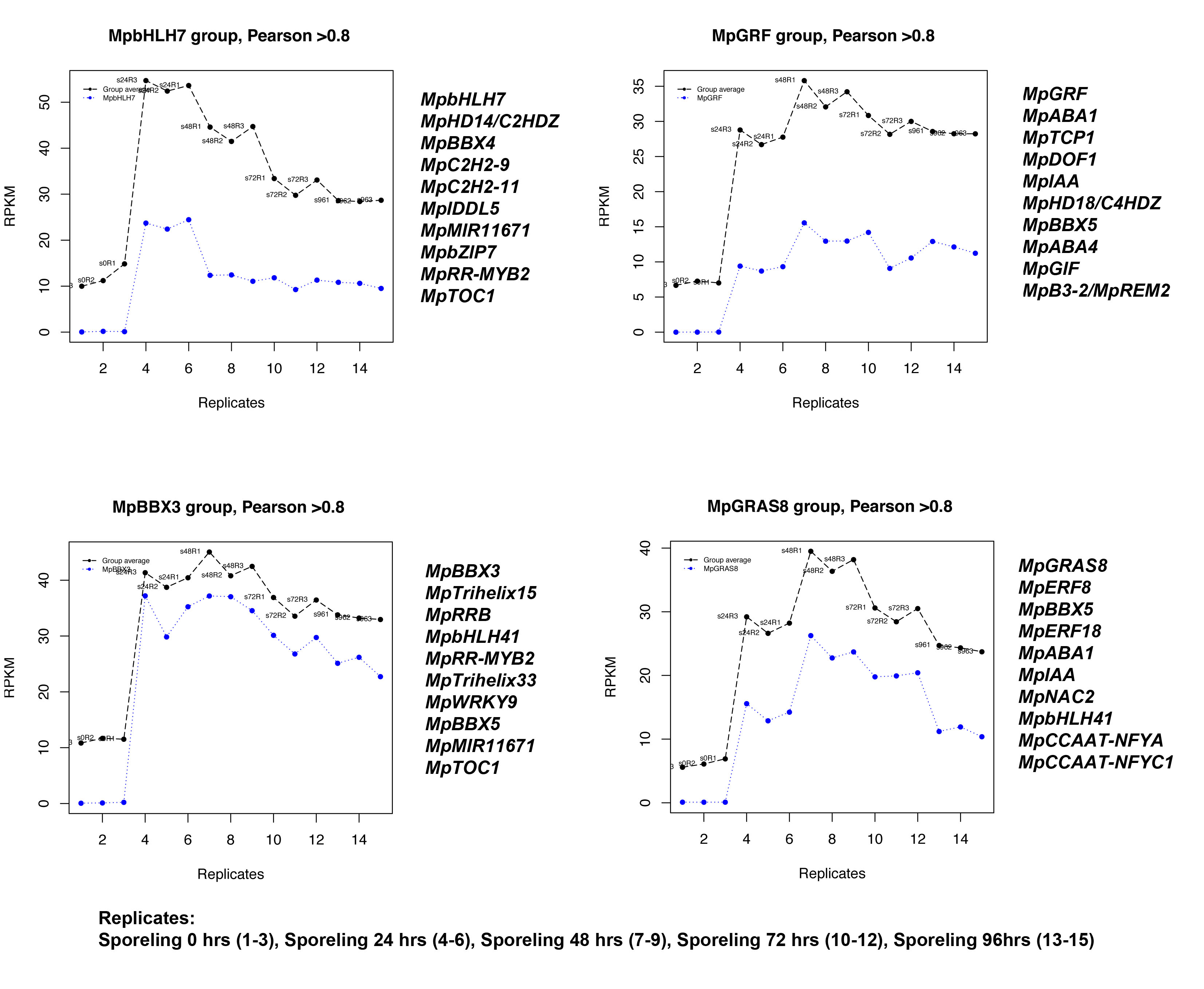

Supplement: FIGURE S2 — Identification of co-expression groups of 24-h enriched TFs in M. polymorpha sporelings. RPKM values are shown for all replicates from 0 to 96 h. [file Image_2.JPEG]

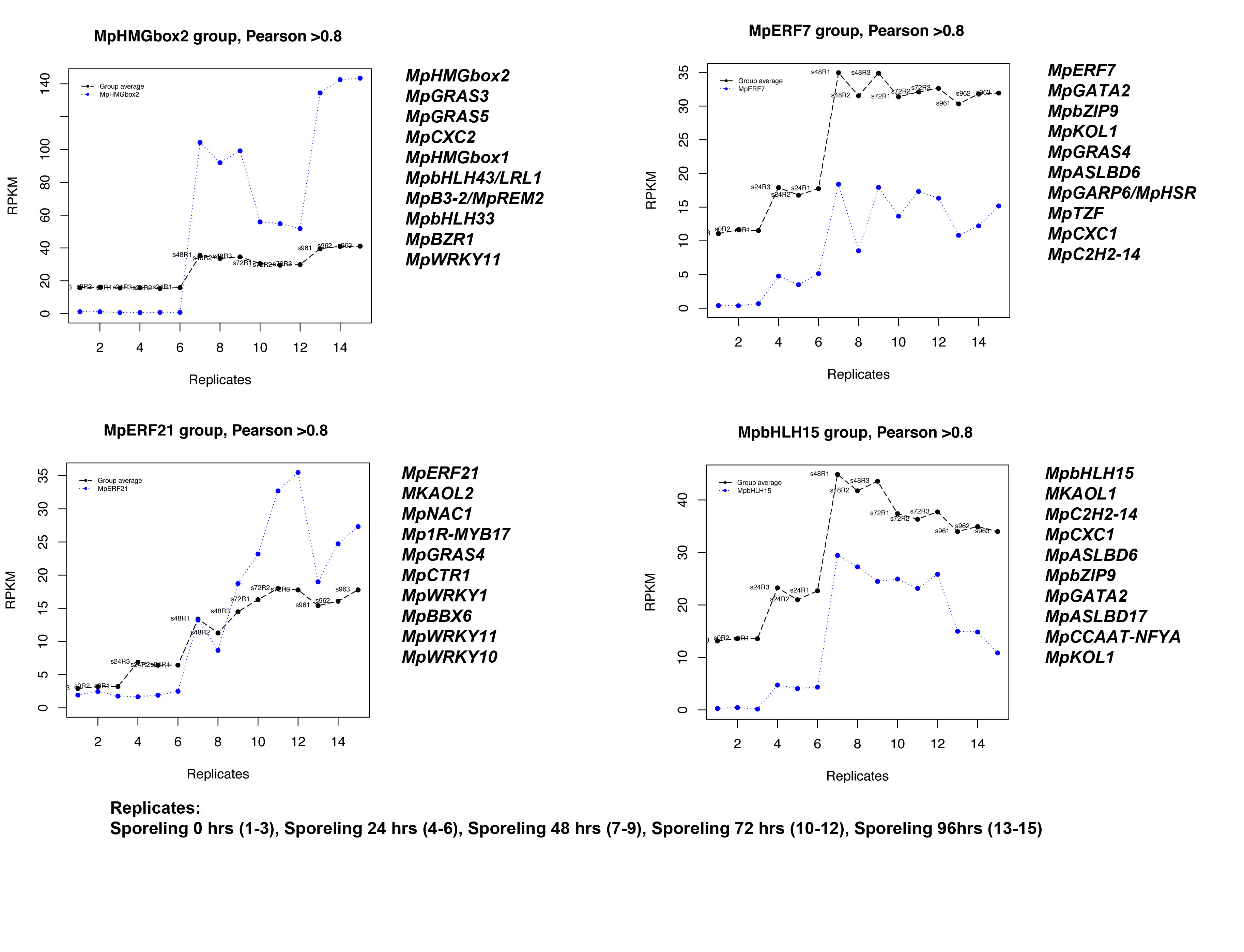

Supplement: FIGURE S3 — Identification of co-expression groups of 48-h enriched TFs M. polymorpha sporelings. RPKM values are shown for all replicates from 0 to 96 h. [file Image_3.JPEG]

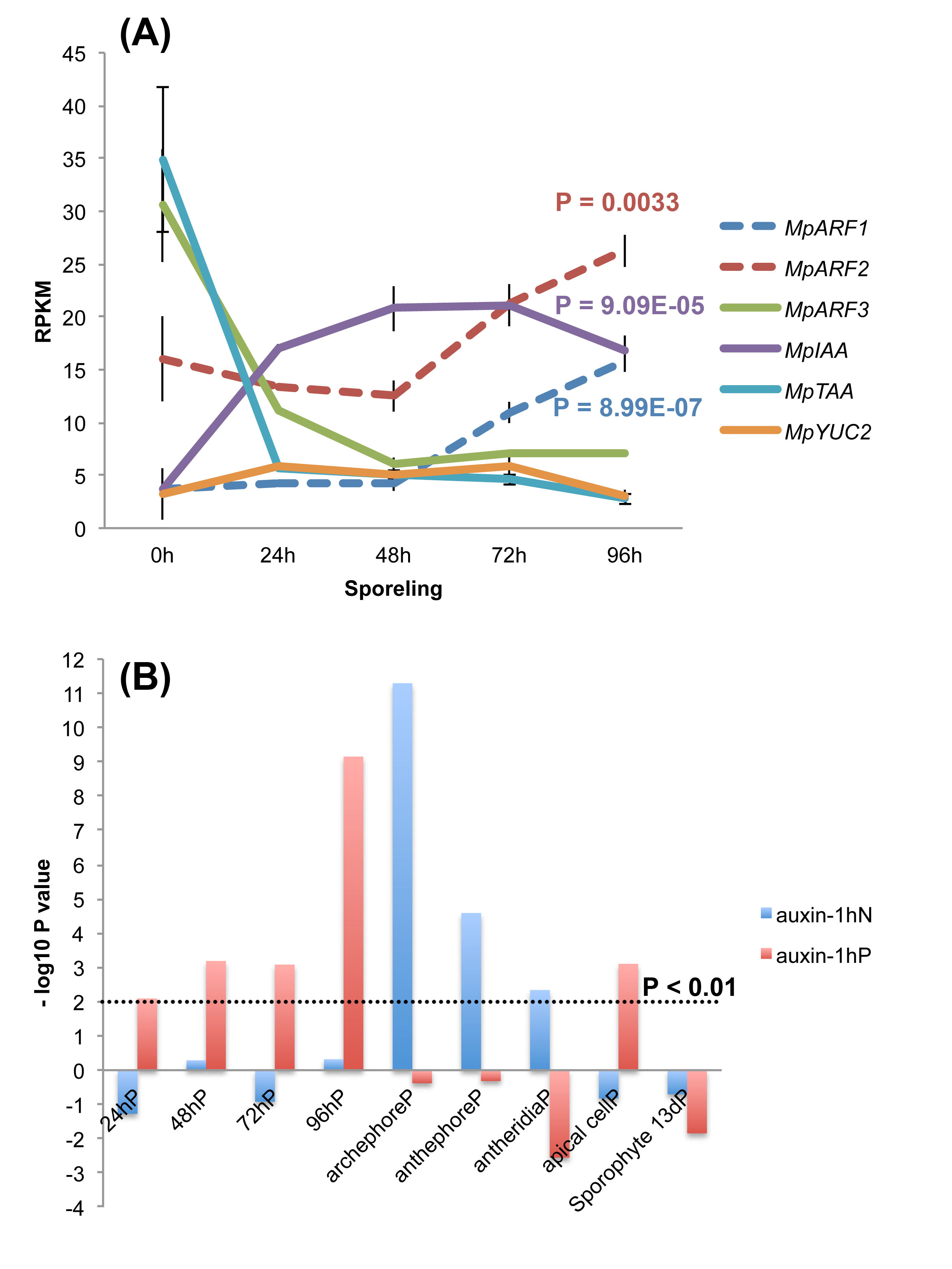

Supplement: FIGURE S4 — Auxin-signaling and biosynthetic gene expression during sporeling development. (A) RPKM averages of MpARF1, MpARF2, MpARF3, MpIAA, MpTAA, and MpYUC2 during the first four days of sporeling development. Error bars indicate SD. Upregulation of MpARF1 and MpARF2 and downregulation of MpIAA at 96 h are statistically supported. P-values marked are from edgeR analysis. (B) Enrichment analysis of auxin upregulated and downregulated genes probed against multiple developmental RNA-Seq libraries obtained from DGE analysis. The tissue with the highest resemblance to the auxin-upregulated transcriptome is 96-h sporelings. [file Image_4.JPEG]

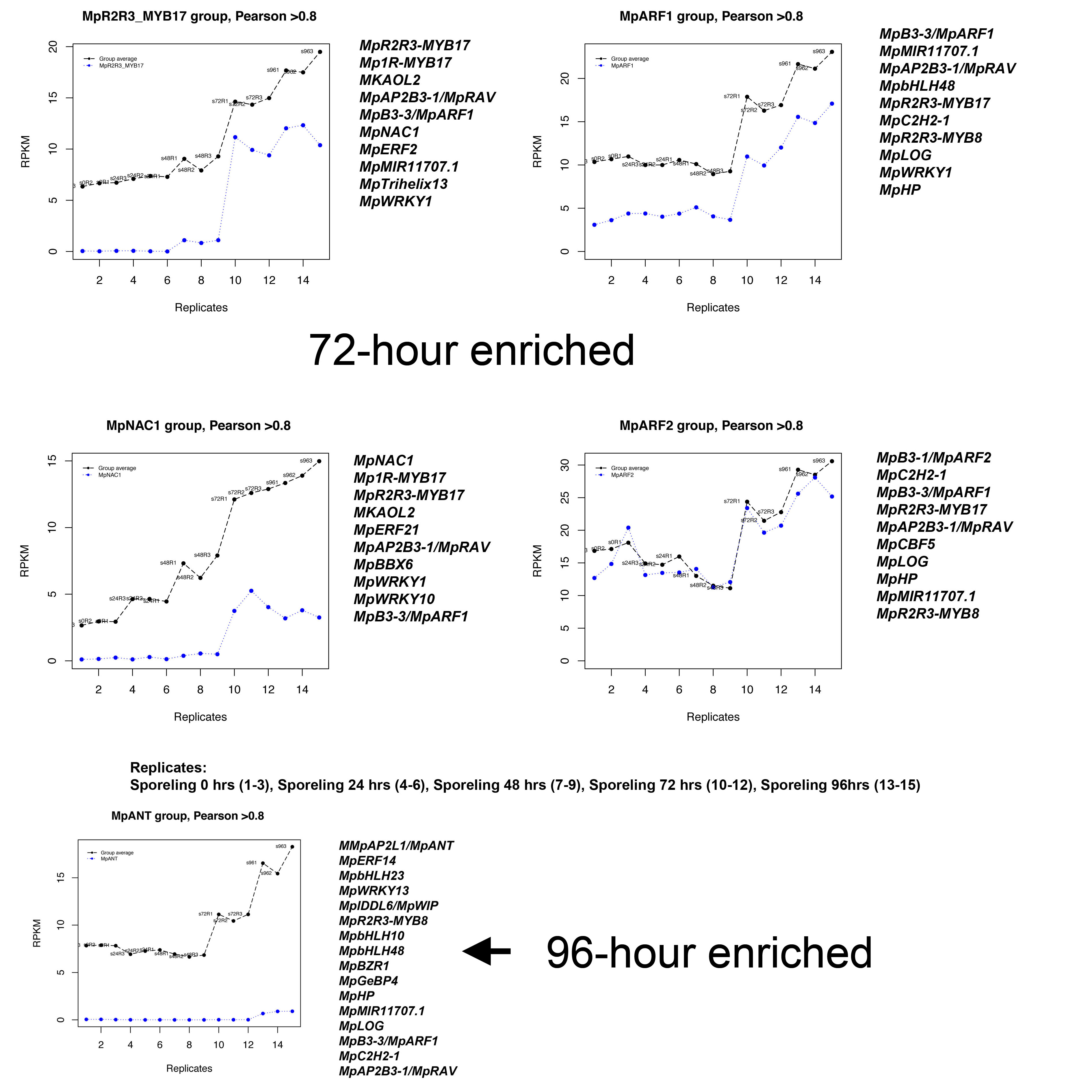

Supplement: FIGURE S5 — Identification of co-expression groups of 72 and 96-h enriched TFs M. polymorpha sporelings. RPKM values are shown for all replicates from 0 to 96 h. [file Image_5.JPEG]

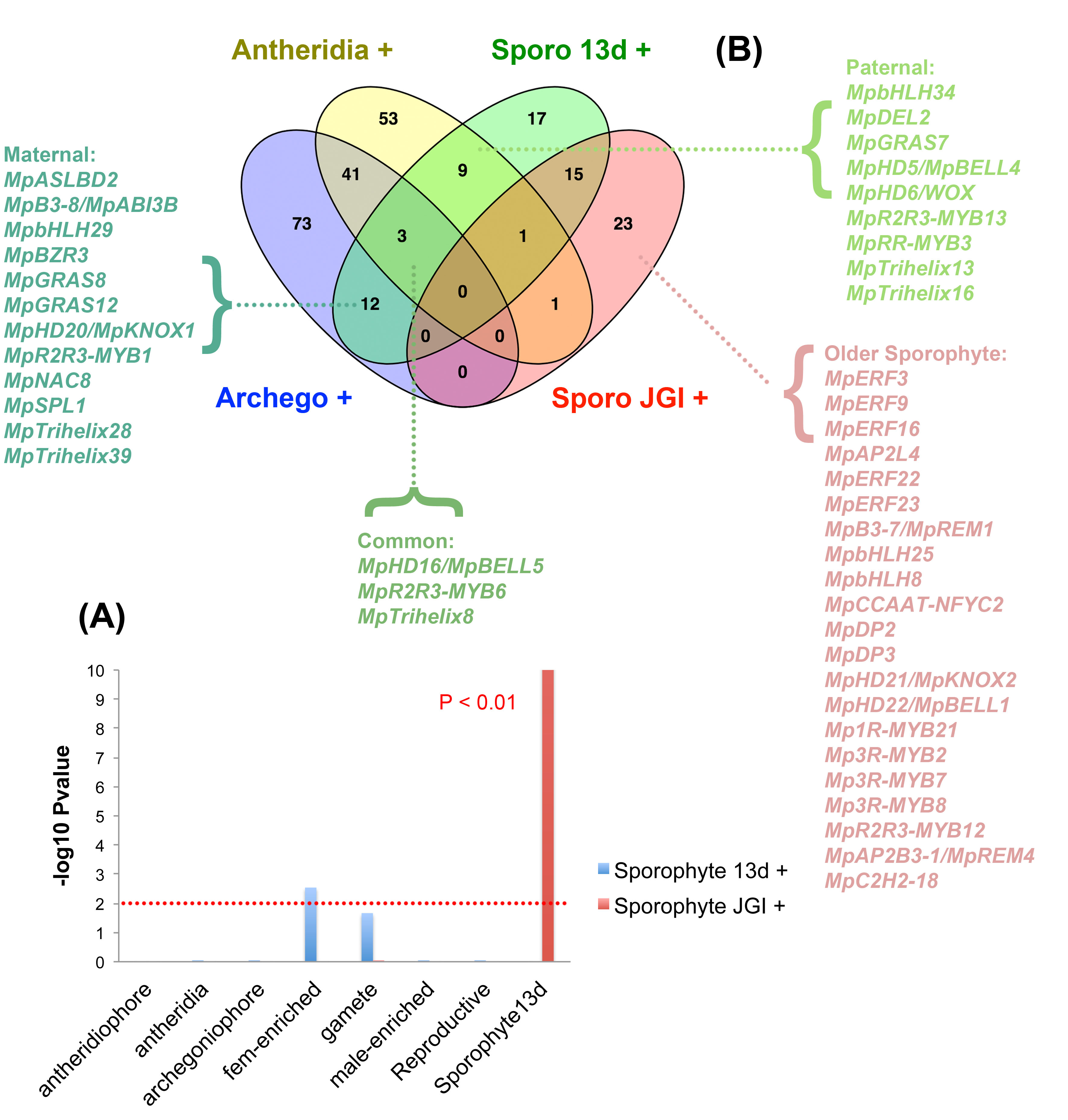

Supplement: FIGURE S6 — Sporophytic gene expression in M. polymorpha. (A) Enrichment analysis of 13-day and JGI manually dissected sporophytes shows significant enrichment between the transcriptomes. Genes classified as female-enriched (Supplementary Table S10) are also statistically represented in 13-day sporophyte transcriptomes. (B) Venn Diagram of shared TFs between antheridia, archegoniophore, 13-day and JGI sporophytes. Putative maternal, paternal and common TFs continuing expression in 13-day sporophytes are annotated. The + symbol indicates upregulation (logFC > 0). [file Image_6.JPEG]

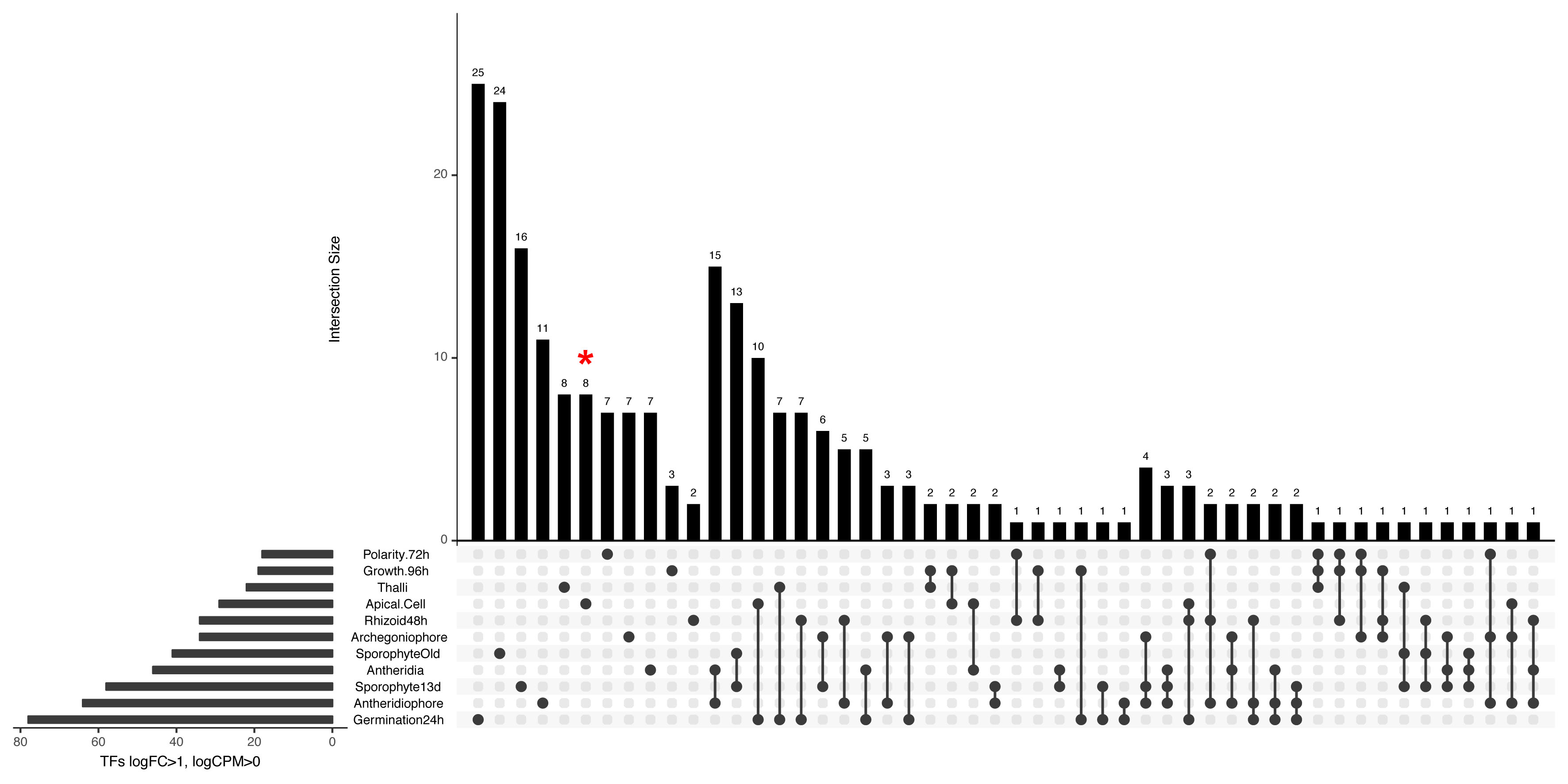

Supplement: FIGURE S7 — UpSet diagram of tissue-specific TF enrichment at logFC > 1. Red asterisk shows eight uniquely upregulated TFs in apical cells at logFC > 1 compared to other tissues. [file Image_7.JPEG]

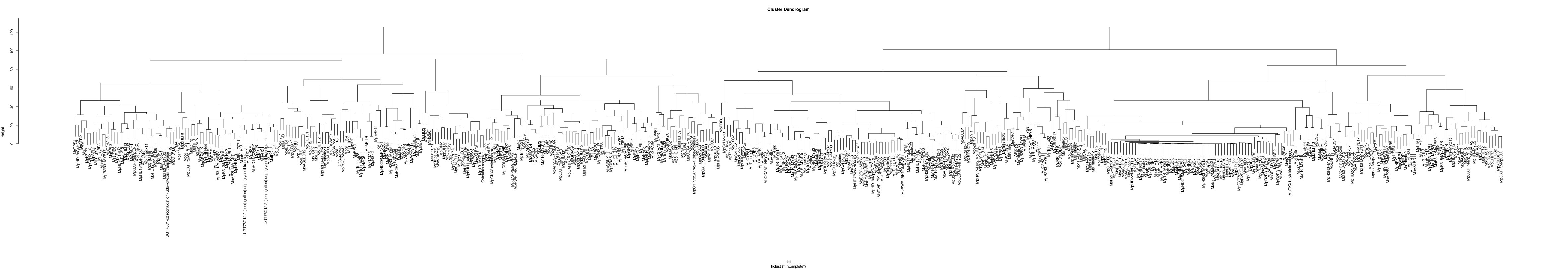

Supplement: FIGURE S8 — Distance dendrogram generated with hclust (R Version 2.3.2) of all annotated M. polymorpha TFs (Supplementary Table S1) in 11 tissue libraries using RPKM > 1 as a threshold. [file Image_8.JPEG]

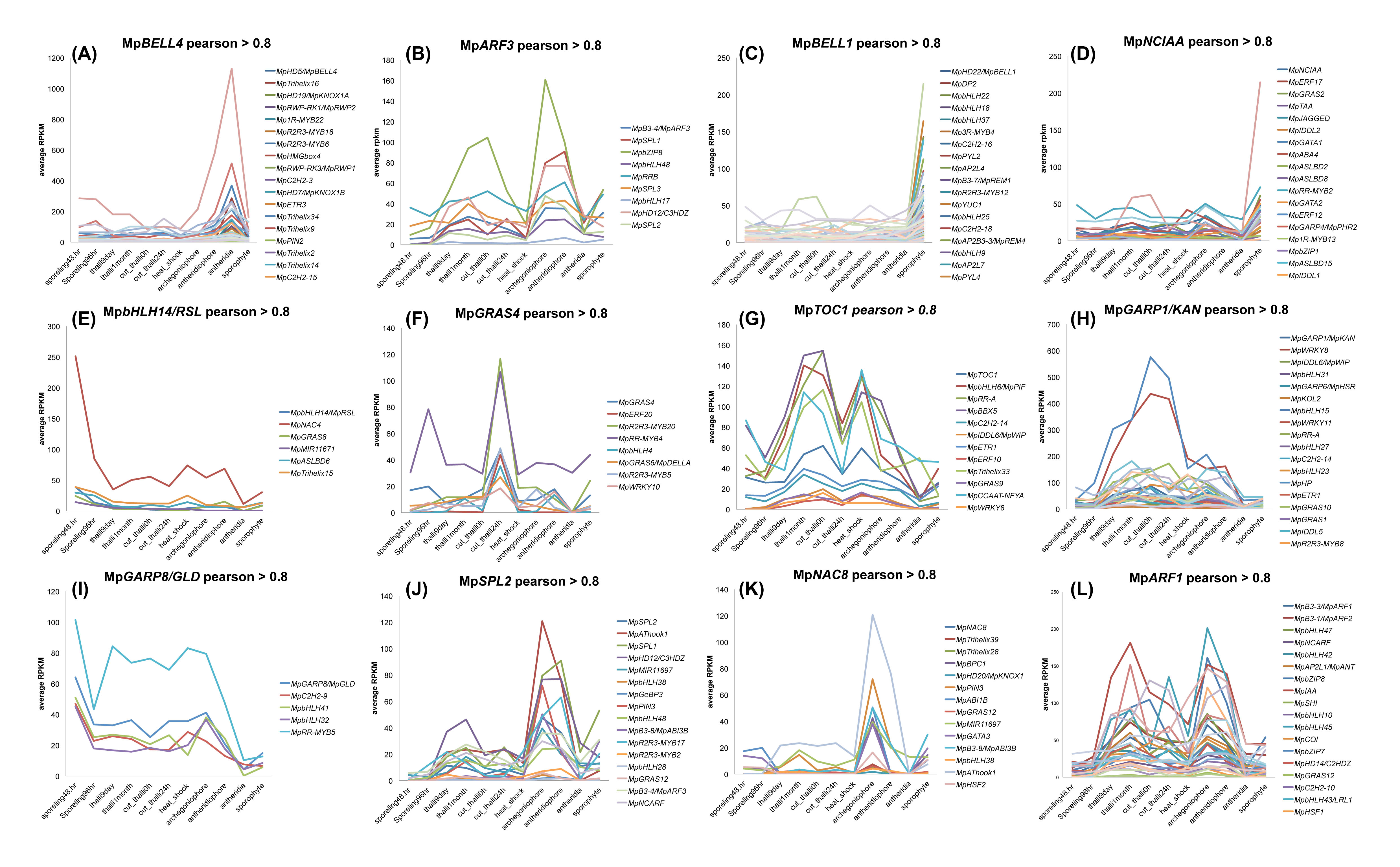

Supplement: FIGURE S9 — RPKM dynamics of TF/response/biosynthetic genes obtained in our analysis. Pearson coefficients above 0.8 were used to define the MpBELL4 (A), MpARF3 (B), MpBELL1 (C), MpNCIAA (D), MpbHLH14/RSL (E), MpGRAS4 (F), MpTOC1 (G), MpGARP1/KAN (H), MpGARP8/GLD (I), MpSPL2 (J), MpNAC8 (K), and MpARF1 (L) coxpression groups. Accompanying TFs are indicated at the left of each graph. [file Image_9.JPEG]

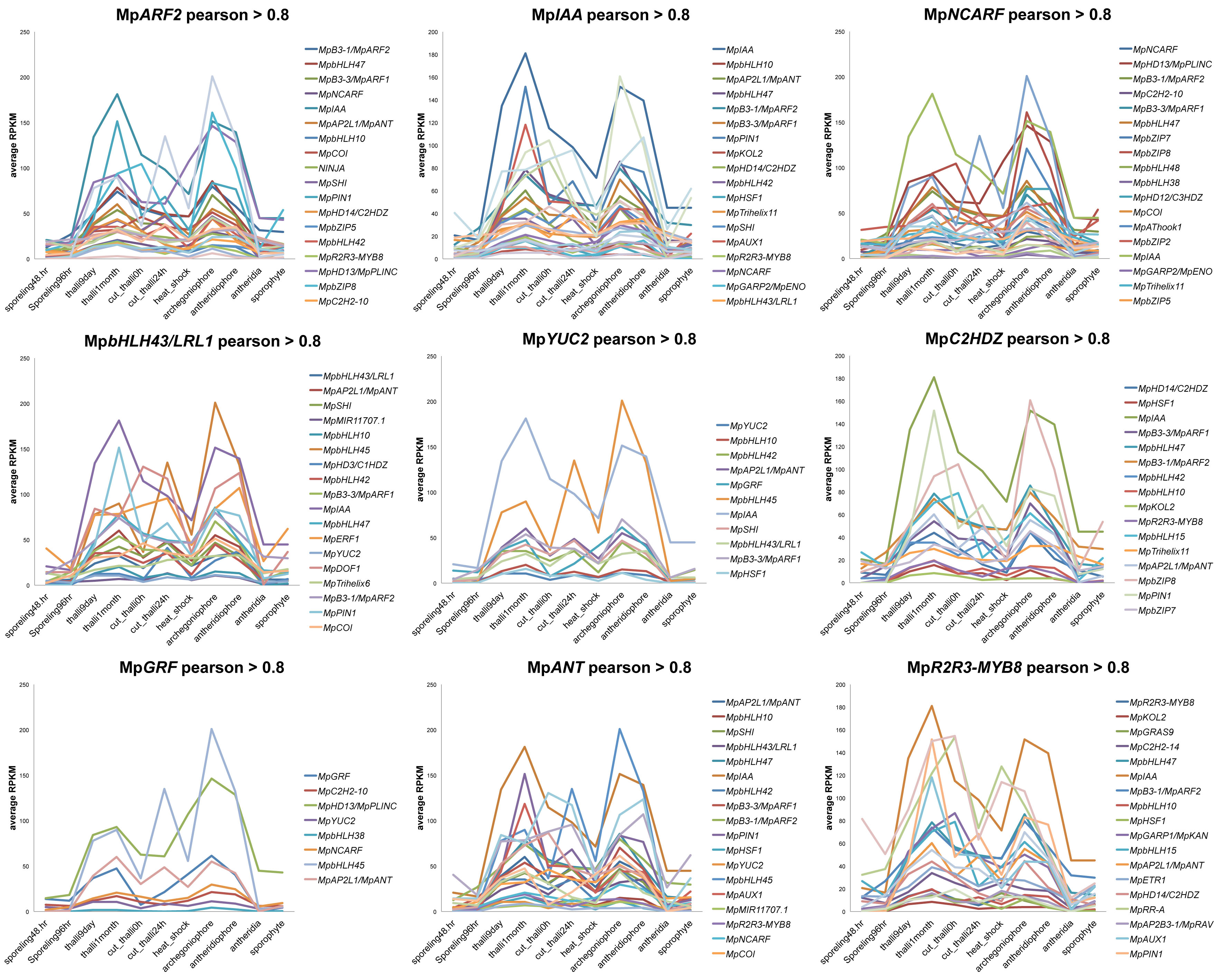

Supplement: FIGURE S10 — RPKM dynamics of the putative auxin co-expression group genes. [file Image_10.JPEG]

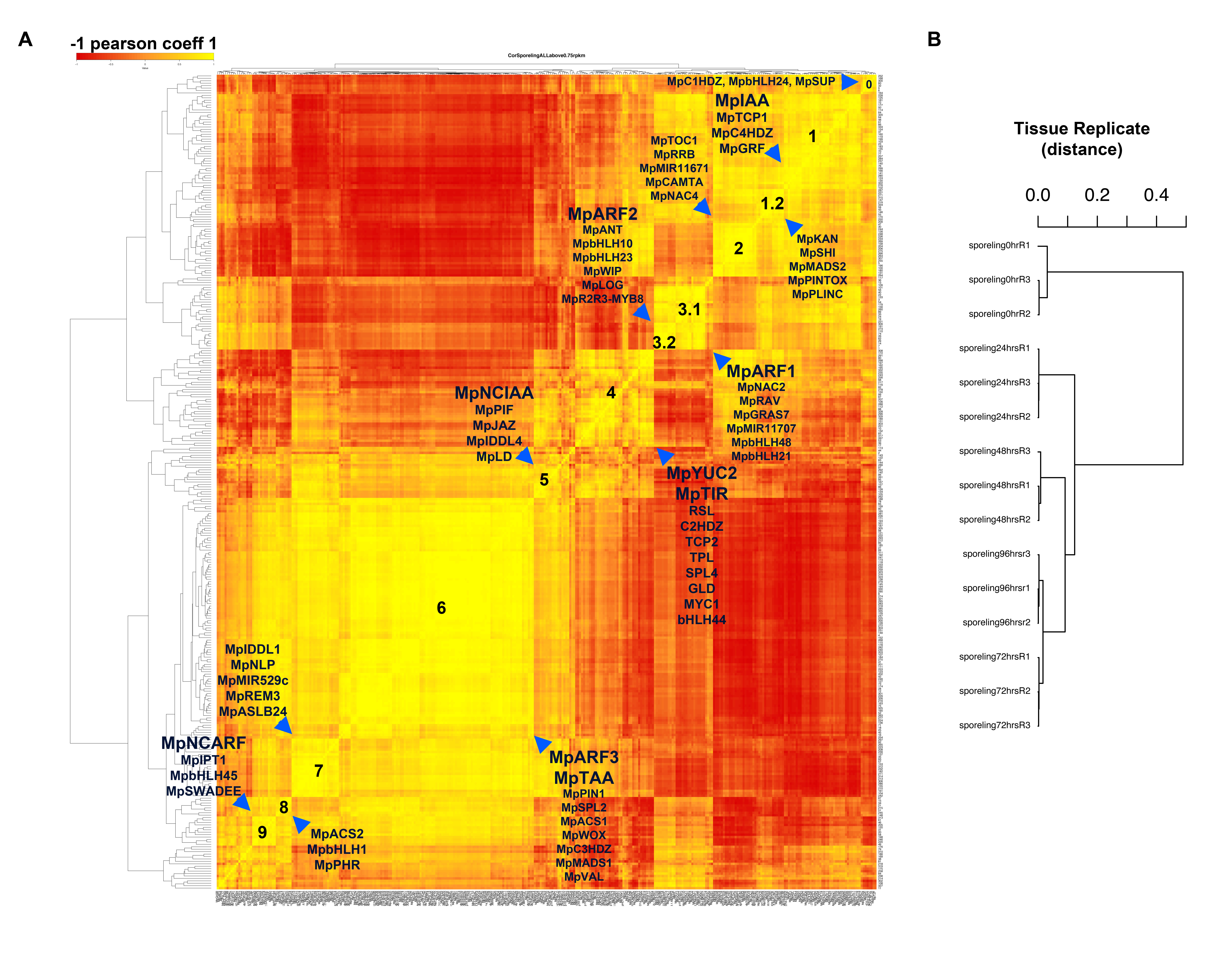

Supplement: FIGURE S11 — (A) Heatmap of Pearson coefficient matrix of all annotated M. polymorpha TFs, MIR precursors and putative hormonal genes (Supplementary Table S16) RPKM values above 0.75 (to include MpANT) in sporeling tissues. Putative groups were delimited by sets of genes sharing coefficients above 0.8. Genes putatively involved in auxin biosynthesis, perception, signaling and transport are mapped, together with accompanying partners in smaller fonts. (B) Distance dendrogram of tissue libraries used in the analysis. Average replicate RPKM values were used per library. Scale for heatmap indicates Pearson coefficients. [file Image_11.JPEG]

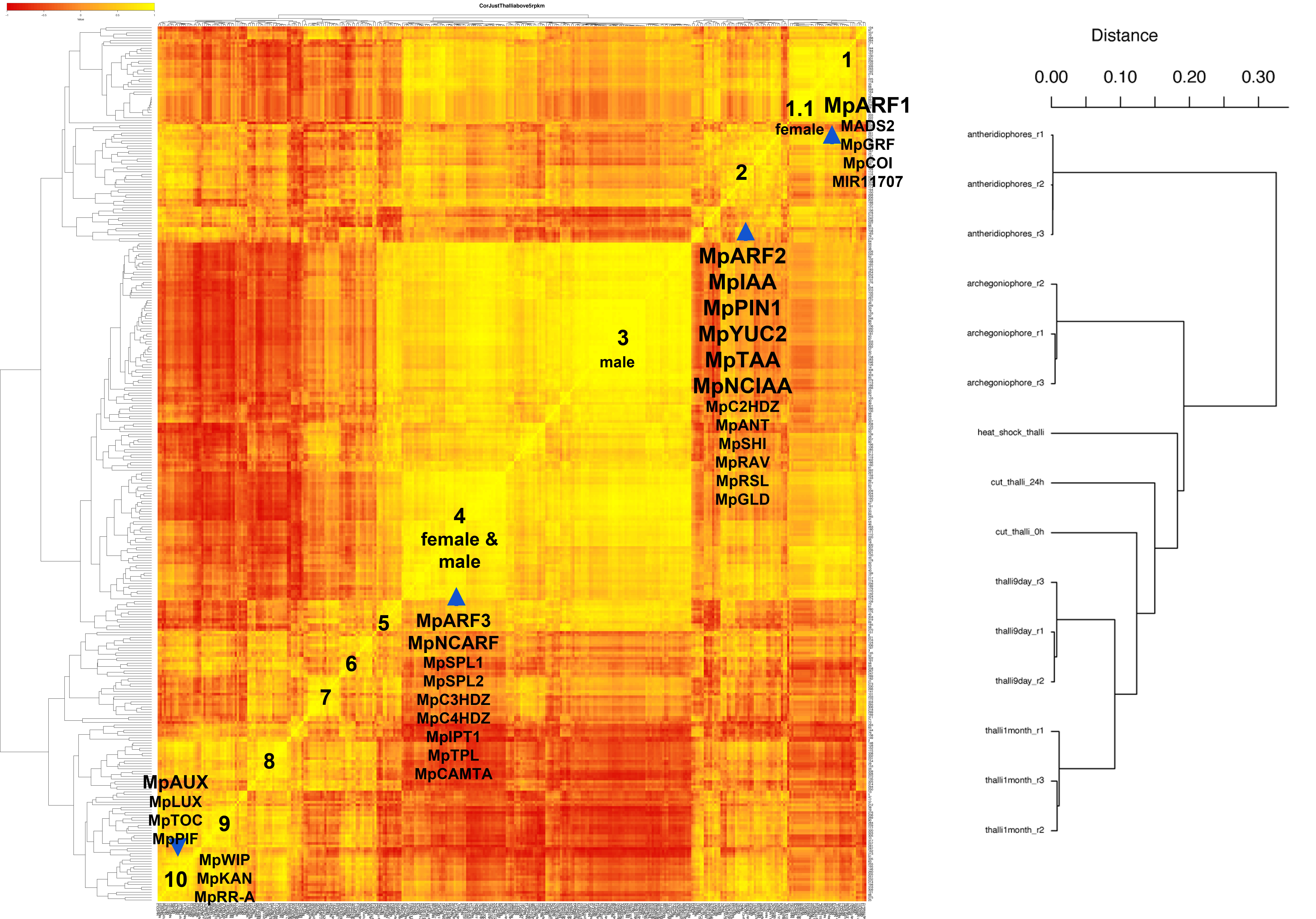

Supplement: FIGURE S12 — (A) Heatmap of Pearson coefficient matrix of all annotated M. polymorpha TFs, MIR precursors and putative hormonal genes with RPMK values above 5 (Supplementary Table S17) in thalli and gametangiophores. Putative groups were delimited by sets of genes sharing coefficients above 0.8. Genes putatively involved in auxin biosynthesis, perception, signaling and transport are mapped, together with accompanying partners in smaller fonts. (B) Distance dendrogram of tissue libraries used in the analysis. Average replicate RPKM values were used per library. Scale for heatmap indicates Pearson coefficients. [file Image_12.JPEG]

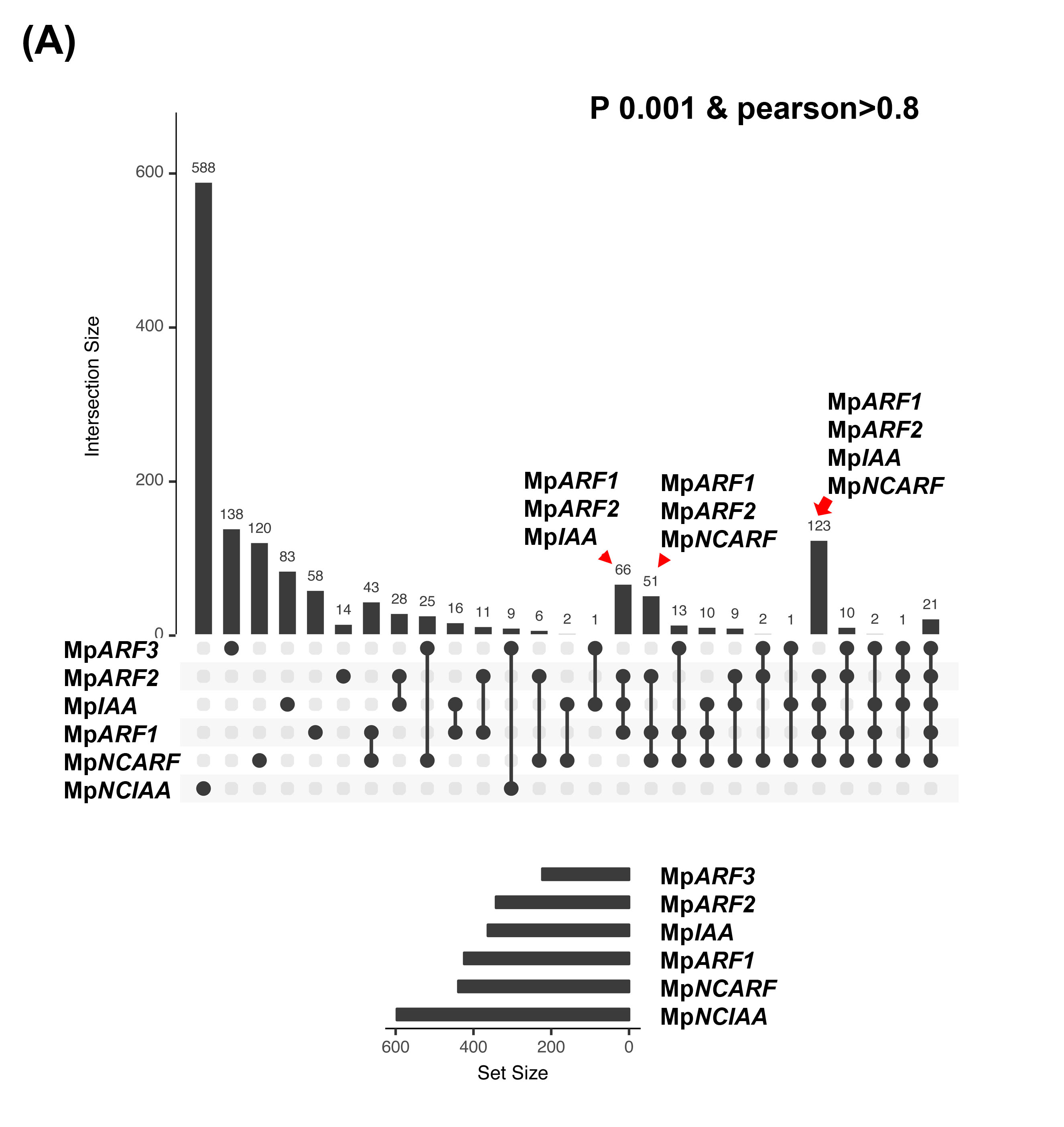

Supplement: FIGURE S13 — (A) UpSet diagram showing shared co-expressed genes (cut-offs indicated) between most PBI-containing genes in M. polymorpha. Numbers of genes exclusive or shared between groups are shown. Co-expression analyses were performed for all expressed genes in our RPKM matrix. [file Image_13.JPEG]

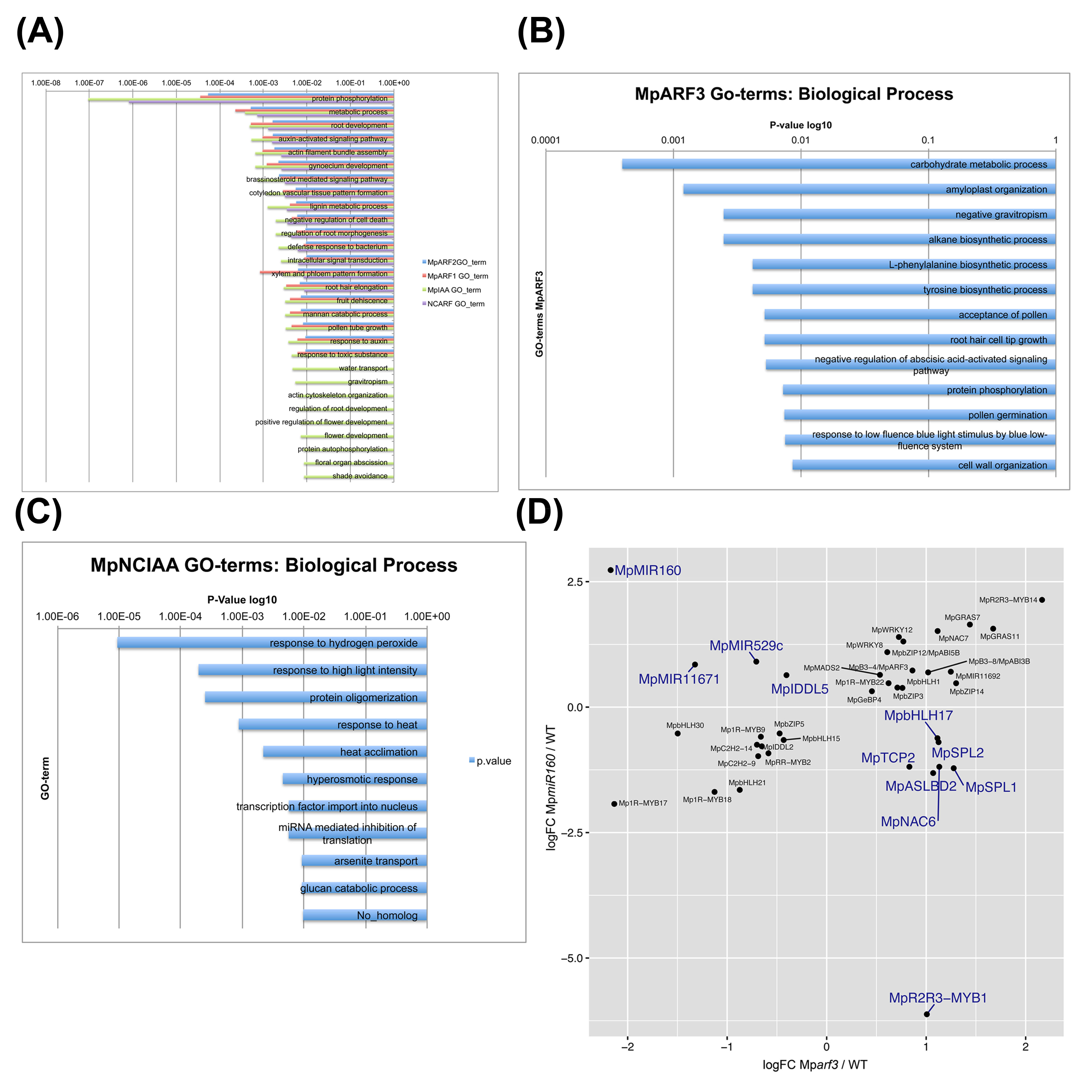

Supplement: FIGURE S14 — (A) Significant GO-terms (Biological Process) found in the MpARF1, MpARF2, MpNCARF, and MpIAA co-expression groups. X-axis indicates P-Values. (B) Significant GO-terms (Biological Process) found in the MpARF3 co-expression group. X-axis indicates P-values. (C) Significant GO-terms (Biological Process) found in the MpNCIAA co-expresion group. X-axis indicates P-values. (D) TFs differentially expressed in Mparf3 and MpmiR160 transcriptomes using DESeq2 (P < 0.01 cut-off). Genes in blue are consistent with MpmiR160-dependent repression of MpARF3. [file Image_14.JPEG]
